# Elucidating the Risk of Colorectal Cancer for Variants in Hereditary Colorectal Cancer Genes

**DOI:** 10.1053/j.gastro.2023.06.032

**Published:** 2023-07-14

**Authors:** KHALID MAHMOOD, MINTA THOMAS, QU CONGHUI, GECCO-CCFR CONSORTIUM, LI HSU, DANIEL D. BUCHANAN, ULRIKE PETERS

**Affiliations:** Colorectal Oncogenomics Group, Department of Clinical Pathology, The University of Melbourne, Parkville, Victoria, Australia, and University of Melbourne Center for Cancer Research, Victorian Comprehensive Cancer Center, Parkville, Victoria, Australia, and Melbourne Bioinformatics, The University of Melbourne, Parkville, Victoria, Australia; Public Health Sciences Division, Fred Hutchinson Cancer Center, Seattle, Washington; Public Health Sciences Division, Fred Hutchinson Cancer Center, Seattle, Washington; Public Health Sciences Division, Fred Hutchinson Cancer Center, Seattle, Washington, and Department of Biostatistics, University of Washington, Seattle, Washington; Colorectal Oncogenomics Group, Department of Clinical Pathology, The University of Melbourne, Parkville, Victoria, Australia, and University of Melbourne Center for Cancer Research, Victorian Comprehensive Cancer Center, Parkville, Victoria, Australia, and Genomic Medicine and Family Cancer Clinic, The Royal Melbourne Hospital, Parkville, Victoria, Australia; Public Health Sciences Division, Fred Hutchinson Cancer Center, Seattle, Washington, and Department of Epidemiology, University of Washington, Seattle, Washington

An important subset of colorectal cancer (CRC) is caused by rare pathogenic variants in more than 20 high-risk genes,^1–3^ The National Comprehensive Cancer Network clinical practice guidelines (2022) recommend that physicians consider multigene panel testing for these high-risk genes in all newly diagnosed CRC patients^2,3^ to identify carriers of pathogenic variants and promote testing of family members who may also be carriers and would benefit from increased screening for CRC prevention. However, clinical challenges remain, such as (1) understanding the risk of CRC associated with individual variants within high-risk genes and (2) for recessively inherited CRC genes, where both alleles of the gene are defective (biallelic) because of the same pathogenic variant (homozygous carriers) or 2 different pathogenic variants (compound heterozygote carriers), understanding if CRC risk is increased if only 1 pathogenic variant is present (monoallelic carriers). Research addressing these challenges will improve clinical actionability regarding the intensity of screening and surveillance for carriers.

We combined genetic data from 58,998 CRC-affected individuals and 71,171 control individuals of European ancestry from 3 major CRC consortia, namely, the Genetics and Epidemiology of Colorectal Cancer Consortium (GECCO), the Colorectal Cancer Transdisciplinary Study (CORECT), and the Colon Cancer Family Registry (CCFR)^1^ (Supplementary Table 1). To enable analysis of rare genetic variants in this dataset, we used the largest available imputation panel based on whole-genome sequencing data from 97,256 samples in the National Heart, Lung, and Blood Institute Trans-Omics for Precision Medicine (TOPMed) study^4^ to impute variants into genome-wide array data for CRC-affected case and control individuals. We examined the association of variants with a minor allele frequency (MAF) of <0.001 in 22 moderate- to high-penetrance CRC genes.^2^ For the recessive CRC genes *MUTYH, NTHL1, MSH3*, and *MBD4*, we assessed the risk of CRC associated with biallelic or monoallelic carriers. If different variants within a gene increase CRC risk, testing all variants simultaneously can be more powerful; therefore, we conducted gene-based tests using the set-based Mixed-Effects Score Test (MiST).^5^ Detailed methods are provided in the Supplementary Material.

We investigated the association of single variants with CRC by modeling the variants as a log-additive effect, which is a more general model. Two significant variants were identified, the APC c.3920T>A:p.Ile1307Lys variant (odds ration [OR], 1.82; *P* =1.62 × 10^–14^), which is more common in the Ashkenazi Jewish population,^6^ and the c.1187G>A:p.Gly396Asp pathogenic variant in *MUTYH* (OR, 1.28; *P* = 2.17 × 10^–5^) (Table 1). Overall, 24 variants in 11 of 22 high-risk genes had a *P* value of <.01, but aside from the 2 variants in *APC* and *MUTYH*, none surpassed multiple comparison correction (Supplementary Table 2). Applying a recessive model for genes known to act recessive demonstrated that the recessive model was a better fit for *MUTYH* because the ORs were larger and *P* values were lower than for the log-additive model. Biallelic carriers of *MUTYH* c.1187G>A:p.Gly396Asp (OR, 32.1; *P* = 1.41 × 10^–6^) or c.536A>G:p.Tyr179Cys (OR, 16.1; *P* = 0.02) and compound heterozygote carriers of these 2 variants had a substantially increased CRC risk (OR, 58.03; *P* = 4.2 × 10^–4^) (Table 1). Monoallelic carriers of either one of these 2 pathogenic variants in *MUTYH* did not demonstrate an increased risk of CRC. Furthermore, we investigated the association between monoallelic *MUTYH* carriers and CRC risk stratified by the presence or absence of 1 or more first-degree relatives with CRC. There was no evidence that monoallelic *MUTYH* carriers had an increased risk of CRC regardless of a family history of CRC (Supplementary Table 3). See the Supplementary Material for an analysis of monoallelic carriers (Supplementary Table 5) in additional recessive genes and candidate pathway genes (Supplementary Table 6).

Previous studies based on substantially smaller sample sizes have reported an increased risk of CRC for monoallelic carriers of *MUTYH* pathogenic variants, especially in the presence of a first-degree relative with CRC,^7^ although the effect size was small. Accordingly, our study provides strong evidence that biallelic, but not monoallelic, inactivation of the *MUTYH* gene predisposes to CRC. This is further supported by observations that COSMIC tumor mutational signatures (SBS18/SBS36), indicative of defective base excision repair, are present only in biallelic *MUTYH* pathogenic variants carriers but not in CRC tumors of monoallelic *MUTYH* pathogenic variant carriers.^8^ Similarly, monoallelic carriers of either of the 2 common pathogenic variants in *NTHL1*, c.268C>T:p.Gln90Ter or c.859C>T:p.Gln287Ter, were not associated with an increased risk of CRC. Our finding confirms a recent analysis of 5942 individuals with CRC or unexplained polyposis that showed no evidence of an increased risk of CRC in monoallelic *NTHL1* loss of function variant carriers and the absence of SBS30 mutational signature unique to biallelic inactivation of NTHL1.^9^

When we tested the association with CRC risk for all predicted pathogenic variants within a gene using gene-based MiST testing, we found that the combined burden of rare predicted pathogenic variants in the APC (*P* = .0007), *MSH3* (*P* = .0216), and *MLH1* (*P* = .0362) genes increased CRC risk, but these associations were driven by a single rare variant per gene. Exclusion of each of these lead variants resulted in a diminished and nonsignificant signal (Table 1).

Imputation to TOPMed allowed us to test a substantially larger number of genetic variants at higher imputation quality than our previous efforts using the Haplotype Reference Consortium^1^ (26.4 million vs 14.8 million single-nucleotide polymorphisms with MAFs between 0.1% and 1%) (Supplementary Table 4). We note that although sequencing provides the best quality, it has been shown that imputation of rare variants is more reliable than direct genotyping using arrays.^10^ Nevertheless, the TOPMed imputation remains limited for variants with MAFs as low as ~0.01%, and large-scale whole-genome sequencing studies are required to identify those ultrarare genetic variants. Following the lead where this has been successful in cardiometabolic and lung diseases via the TOPMed program, dedicated funding is required to enable a well-powered discovery effort for cancer. The study population is limited to individuals of European descent and needs to be expanded to include other ancestry populations.

This well-powered study provides strong evidence that monoallelic pathogenic variant carriers in *MUTYH*, regardless of family history of CRC, as well as other recessive-acting genes, such as *NTHL1*, *MSH3*, and *MBD4* do not have an increased CRC risk. Accordingly, intensified surveillance of monoallelic carriers of these recessive genes, which have a relatively high population frequency close to 1%, is not warranted.

## Supplementary Material

1

2

## Figures and Tables

**Table 1.** Imputed Germline Variants in High-Risk CRC Genes and the Association With CRC Risk Based on Individual Variant Level Analysis, Recessive Model Analysis of the 2 Common Pathogenic Variants in the *MUTYH* Gene, and the Set-Based MiST Gene Burden Analysis

Individual variant–level analysis						

Gene	Variant	gnomAD NFE frequency	Imputation accuracy	Case/control individuals	OR (95% CI)	*P* value

*APC*	c.3920T>A, p.Ile1307Lys (rs1801155)	6.4 × 10^−4^ (AJ, 0.036)	0.948	519/306	1.82 (1.56–2.12)	1.62 × 10^−14a^
*MUTYH* ^b^	c.1187G>A, p.Gly396Asp (rs36053993)	5.4 × 10^−3^	0.995	747/769	1.28 (1.15–1.42)	2.17 × 10^−5a^
*MUTYH* ^b^	c.536A>G, Tyr179Cys (rs34612342)	2.3 × 10^−3^	0.872	232/239	1.35 (1.11–1.67)	3.5 × 10^−3^
*NTHL1*	c.268C>T p.Gln90Ter (rs150766139)	1.8 × 10^−3^	0.758	195/224	1.11 (0.89–1.39)	0.54
*NTHL1*	c.859C>T p.Gln287Ter (rs146347092)	3.6 × 10^−4^	0.558	15/29	0.43 (0.18–0.90)	0.6

Recessive analysis							

		rs34612342 (c.536A>G, Tyr179Cys or Y179C)					
	
		TT		TC		CC	
			
*MUTYH*		Case/control individuals	OR (95% CI)	Case/control individuals	OR (95% CI)	Case/control individuals	OR (95% CI)

rs36053993 (c.1187G>A, Gly396Asp or G396D)	CC	58,049/70,475	1.00 (Reference)	194/237	1.13 (0.91–1.40) *P* = .26	8/1	16.1 (1.42–181.72) *P* = .02
	CT	665/766	1.09 (0.98–1.22) *P* = .12	30/1	58.03 (6.1–552.8) *P* = 4.2 × 10^−4^	0/0	—
	TT	52/2	32.1 (7.8–131.5) *P* = 1.4 × 10^−6^	0/0	—	0/0	—

Gene set-based MiST analysis					

Gene	Number of variants	Lead variant	OR (95% CI)	MiST *P* value	MiST *P* value^c^ (minus lead variant)

*APC*	15	c.3920T>A, p.Ile1307Lys (rs1801155)	1.82 (1.56–2.12)	0.0007	.85
*MLH1*	7	c.1321G>A, p.Ala441Thr (rs63750365)	1.55 (1.02–2.37)	0.0362	.16
*MSH3* ^d^	12	c.2262A>G, p.Ile754Met (rs200819607)	0.406 (0.184–0.897)	0.0216	.21

NOTE. The lead variant is the most associated variant at the locus. The reference single-nucleotide polymorphism cluster ID is based on the National Center for Biotechnology Information dbSNP Build 150. Alleles are on the positive strand. *P* values are based on fixed-effects inverse variance-weighted meta-analysis. For recessive analysis, OR is the OR estimate for the risk allele. *P* values reported in this section are based on pooled data analysis. Estimates were adjusted for age, sex, and genom-ewide association study genotyping platform. For set-based MiST analysis, genetic variants in each gene were restricted to missense, stop-gained, frameshift, and splice site variants with a minor allele count of >10, an MAF of <5%, and an imputation *R*^2^ of >0.3. CADD and REVEL prediction scores were included as continuous functional weights: 1 if CADD > 20 or REVEL > 0.5.

AJ, Ashkenazi Jewish gnomAD frequency; CI, confidence interval; NFE, non-Finnish European gnomAD frequency.

a Statistically significant *P* value based on Bonferroni correction to account for multiple comparisons.

b The number of carriers among case or control individuals for each of these 2 pathogenic variants includes counts of all carriers regardless of whether they occur as heterozygous/monoallelic, compound heterozygous, or homozygous/biallelic carriers.

c Indicates MiST *P* value excluding the lead variant.

d Genes inherited in an autosomal recessive pattern.

## Abbreviations used in this paper:

CADD: Combined Annotation Dependent Depletion
CRC: colorectal cancer
gnomAD: Genome Aggregation Database
MAF: minor allele frequency
MiST: Mixed-Effects Score Test
OR: odds ratio
REVEL: Rare Exome Variant Ensemble Learner
SNP: single nucleotide polymorphism
TOPMed: Trans-Omics for Precision Medicine

## Acknowledgments

The GECCO-CCFRC Consortium includes Xiaoliang Wang,^1^ Jeroen R. Huyghe,^1^ Jihoon E. Joo,^2,3^ Peter Georgeson,^2,3^ Volker Arndt,^4^ Sonja I. Berndt,^5^ Stéphane Bézieau,^6^ Stephanie A. Bien,^1^ D. Timothy Bishop,^7^ Hermann Brenner,^4,8,9^ Stefanie Brezina,^10^ Andrea N. Burnett-Hartman,^11^ Peter T. Campbell,^12^ Graham Casey,^13^ Sergi Castellví-Bel,^14^ Andrew T. Chan,^15,16,17,18,19,20^ Jenny Chang-Claude,^21,22^ Xuechen Chen,^4,23^ David V. Conti,^24^ Chiara Cremolini,^25,26^ Brenda Diergaarde,^27^ Jane C. Figueiredo,^28^ Liesel M. FitzGerald,^29^ Manuela Gago-Dominguez,^30,31^ Steven Gallinger,^32^ Graham G. Giles,^33,34,35^ Andrea Gsur,^10^ Marc J. Gunter,^36^ Jochen Hampe,^37^ Heather Hampel,^38^ Tabitha A. Harrison,^1,39^ Michael Hoffmeister,^4^ Temitope O. Keku,^40^ Anshul Kundaje,^41,42^ Loic Le Marchand,^43^ Heinz-Josef Lenz,^44^ Christopher I. Li,^1^ Li Li,^45^ Yi Lin,^1^ Annika Lindblom,^46,47^ Victor Moreno,^48,49,50,51^ Neil Murphy,^36^ Polly A. Newcomb,^1,52^ Christina C. Newton,^53^ Mireia Obón-Santacana,^48,49^ Shuji Ogino,^18,19,54,55^ Rish K. Pai,^56^ Julie R. Palmer,^57,58^ Rachel Pearlman,^38^ Paul D.P. Pharoah,^59^ Amanda I. Phipps,^1,60^ Elizabeth A. Platz,^61^ John D. Potter,^1,62^ Gad Rennert,^63,64,65^ Lori C. Sakoda,^1,66^ Clemens Schafmayer,^67^ Stephanie L. Schmit,^68,69^ Robert E. Schoen,^70^ Martha L. Slattery,^71^ Zsofia K. Stadler,^72^ Robert S. Steinfelder,^1^ Stephen N. Thibodeau,^73^ Cornelia M. Ulrich,^74^ Caroline Y. Um,^53^ Franzel J.B. van Duijnhoven,^75^ Bethany Van Guelpen,^76,77^ Kala Visvanathan,^61^ Pavel Vodicka,^78,79,80^ Ludmila Vodickova,^78,79,80^ Veronika Vymetalkova,^78,79,80^ Stephanie J. Weinstein,^5^ Emily White,^81,82^ Ingrid M. Winship,^83,84^ Alicja Wolk,^85,86^ Stephen B. Gruber,^87^ and Mark A. Jenkins^34^; from the ^1^Public Health Sciences Division, Fred Hutchinson Cancer Center, Seattle, Washington; ^2^Colorectal Oncogenomics Group, Department of Clinical Pathology, The University of Melbourne, Parkville, Victoria, Australia; ^3^University of Melbourne Center for Cancer Research, Victorian Comprehensive Cancer Center, Parkville, Victoria, Australia; ^4^Division of Clinical Epidemiology and Aging Research, German Cancer Research Center, Heidelberg, Germany; ^5^Division of Cancer Epidemiology and Genetics, National Cancer Institute, National Institutes of Health, Bethesda, Maryland; ^6^Service de Génétique Médicale, Centre Hospitalier Universitaire Nantes, Nantes, France; ^7^Leeds Institute of Medical Research at St. James’s, University of Leeds, Leeds, United Kingdom; ^8^Division of Preventive Oncology, German Cancer Research Center and National Center for Tumor Diseases, Heidelberg, Germany; ^9^German Cancer Consortium, German Cancer Research Center, Heidelberg, Germany; ^10^Institute of Cancer Research, Department of Medicine I, Medical University Vienna, Vienna, Austria; ^11^Institute for Health Research, Kaiser Permanente Colorado, Denver, Colorado; ^12^Department of Epidemiology and Population Health, Albert Einstein College of Medicine, Bronx, New York; ^13^Center for Public Health Genomics, University of Virginia, Charlottesville, Virginia; ^14^Gastroenterology Department, Hospital Clínic, Institut d’Investigacions Biomèdiques August Pi i Sunyer, Centro de Investigación Biomédica en Red de Enfermedades Hepáticas y Digestivas, University of Barcelona, Barcelona, Spain; ^15^Division of Gastroenterology, Massachusetts General Hospital and Harvard Medical School, Boston, Massachusetts; ^16^Channing Division of Network Medicine, Brigham and Women’s Hospital and Harvard Medical School, Boston, Massachusetts; ^17^Clinical and Translational Epidemiology Unit, Massachusetts General Hospital and Harvard Medical School, Boston, Massachusetts; ^18^Broad Institute of Harvard and Massachusetts Institute of Technology, Cambridge, Massachusetts; ^19^Department of Epidemiology, Harvard T.H. Chan School of Public Health, Harvard University, Boston, Massachusetts; ^20^Department of Immunology and Infectious Diseases, Harvard T.H. Chan School of Public Health, Harvard University, Boston, Massachusetts; ^21^Division of Cancer Epidemiology, German Cancer Research Center, Heidelberg, Germany; ^22^University Medical Center Hamburg-Eppendorf, University Cancer Center Hamburg, Hamburg, Germany; ^23^Medical Faculty Heidelberg, Heidelberg University, Heidelberg, Germany; ^24^Department of Population and Public Health Sciences, Keck School of Medicine, University of Southern California, Los Angeles, California; ^25^Department of Translational Research and New Technologies in Medicine and Surgery, University of Pisa, Pisa, Italy; ^26^Unit of Medical Oncology 2, Azienda Ospedaliero-Universitaria Pisana, Pisa, Italy; ^27^Department of Human Genetics, Graduate School of Public Health, University of Pittsburgh, Pittsburgh, Pennsylvania; ^28^Department of Medicine, Samuel Oschin Comprehensive Cancer Institute, Cedars-Sinai Medical Center, Los Angeles, CA; ^29^Menzies Institute for Medical Research, University of Tasmania, Hobart, Tasmania, Australia; ^30^Fundación Gallega de Medicina Genómica, Grupo de Genética del Cáncer, Instituto de Investigación Sanitaria de Santiago, Complejo Hospitalario Universitario de Santiago, SERGAS, Spain; ^31^University of California San Diego, Moores Cancer Center, La Jolla, CA; ^32^Lunenfeld Tanenbaum Research Institute, Mount Sinai Hospital, University of Toronto, Toronto, Ontario, Canada; ^33^Cancer Epidemiology Division, Cancer Council Victoria, Melbourne, Victoria, Australia; ^34^Center for Epidemiology and Biostatistics, Melbourne School of Population and Global Health, The University of Melbourne, Melbourne, Victoria, Australia; ^35^Precision Medicine, School of Clinical Sciences at Monash Health, Monash University, Clayton, Victoria, Australia; ^36^Nutrition and Metabolism Branch, International Agency for Research on Cancer, World Health Organization, Lyon, France; ^37^Department of Medicine I, University Hospital Dresden, Technische Universität Dresden, Dresden, Germany; ^38^Division of Human Genetics, Department of Internal Medicine, The Ohio State University Comprehensive Cancer Center, Columbus, Ohio; ^39^Department of Epidemiology, University of Washington, Seattle, Washington; ^40^Center for Gastrointestinal Biology and Disease, University of North Carolina, Chapel Hill, North Carolina; ^41^Department of Genetics, Stanford University, Stanford, California; ^42^Department of Computer Science, Stanford University, Stanford, California; ^43^University of Hawaii Cancer Center, Honolulu, Hawaii; ^44^Department of Medicine, University of Southern California, Los Angeles, California; ^45^Department of Family Medicine, University of Virginia, Charlottesville, Virginia; ^46^Department of Clinical Genetics, Karolinska University Hospital, Stockholm, Sweden; ^47^Department of Molecular Medicine and Surgery, Karolinska Institutet, Stockholm, Sweden; ^48^Oncology Data Analytics Program, Bellvitge Biomedical Research Institute (IDIBELL), L’Hospitalet de Llobregat, Barcelona, Spain; ^49^CIBER Epidemiología y Salud Pública, Madrid, Spain; ^50^Department of Clinical Sciences, Faculty of Medicine, University of Barcelona, Barcelona, Spain; ^51^Oncobell Program, Bellvitge Biomedical Research Institute (IDIBELL), L’Hospitalet de Llobregat, Barcelona, Spain; ^52^School of Public Health, University of Washington, Seattle, Washington; ^53^Department of Population Science, American Cancer Society, Atlanta, Georgia; ^54^Program in MPE Molecular Pathological Epidemiology, Department of Pathology, Brigham and Women’s Hospital, Harvard Medical School, Boston, Massachusetts; ^55^Cancer Immunology Program, Dana-Farber/Harvard Cancer Center, Boston, Massachusetts; ^56^Department of Laboratory Medicine and Pathology, Mayo Clinic Arizona, Scottsdale, Arizona; ^57^Boston University School of Public Health, Boston, Massachusetts; ^58^Slone Epidemiology Center, Boston University, Boston, Massachusetts; ^59^Department of Public Health and Primary Care, University of Cambridge, Cambridge, United Kingdom; ^60^Department of Epidemiology, University of Washington, Seattle, Washington; ^61^Department of Epidemiology, Johns Hopkins Bloomberg School of Public Health, Baltimore, Maryland; ^62^Research Center for Hauora and Health, Massey University, Wellington, New Zealand; ^63^Department of Community Medicine and Epidemiology, Lady Davis Carmel Medical Center, Haifa, Israel; ^64^Ruth and Bruce Rappaport Faculty of Medicine, Technion-Israel Institute of Technology, Haifa, Israel; ^65^Clalit National Cancer Control Center, Haifa, Israel; ^66^Division of Research, Kaiser Permanente Northern California, Oakland, California; ^67^Department of General Surgery, University Hospital Rostock, Rostock, Germany; ^68^Genomic Medicine Institute, Cleveland Clinic, Cleveland, Ohio; ^69^Population and Cancer Prevention Program, Case Comprehensive Cancer Center, Cleveland, Ohio; ^70^Departments of Medicine and Epidemiology, University of Pittsburgh Medical Center, Pittsburgh, Pennsylvania; ^71^Department of Internal Medicine, University of Utah, Salt Lake City, Utah; ^72^Department of Medicine, Memorial Sloan Kettering Cancer Center, New York, New York; ^73^Division of Laboratory Genetics, Department of Laboratory Medicine and Pathology, Mayo Clinic, Rochester, Minnesota; ^74^Huntsman Cancer Institute and Department of Population Health Sciences, University of Utah, Salt Lake City, Utah; ^75^Division of Human Nutrition and Health, Wageningen University & Research, Wageningen, the Netherlands; ^76^Department of Radiation Sciences, Oncology Unit, Umeå University, Umeå, Sweden; ^77^Wallenberg Center for Molecular Medicine, Umeå University, Umeå, Sweden; ^78^Department of Molecular Biology of Cancer, Institute of Experimental Medicine of the Czech Academy of Sciences, Prague, Czech Republic; ^79^Institute of Biology and Medical Genetics, First Faculty of Medicine, Charles University, Prague, Czech Republic; ^80^Faculty of Medicine and Biomedical Center in Pilsen, Charles University, Pilsen, Czech Republic; ^81^Public Health Sciences Division, Fred Hutchinson Cancer Research Center, Seattle, Washington; ^82^Department of Epidemiology, University of Washington School of Public Health, Seattle, Washington; ^83^Department of Medicine, The Royal Melbourne Hospital, The University of Melbourne, Parkville, Victoria, Australia; ^84^Genomic Medicine and Family Cancer Clinic, The Royal Melbourne Hospital, Parkville, Victoria, Australia; ^85^Institute of Environmental Medicine, Karolinska Institutet, Stockholm, Sweden; ^86^Department of Surgical Sciences, Uppsala University, Uppsala, Sweden; and ^87^Department of Medical Oncology and Therapeutics Research, City of Hope National Medical Center, Duarte, California.

ASTERISK: We are very grateful to D. Bruno Buecher without whom this project would not have existed. We also thank all those who agreed to participate in this study, including the patients and the healthy control persons, as well as all the physicians, technicians, and students.

Colon Cancer Family Registry (CCFR): The CCFR graciously thanks the generous contributions of their study participants, dedication of study staff, and the financial support from the US National Cancer Institute, without which this important registry would not exist. The authors would like to thank the study participants and staff of the Seattle Colon Cancer Family Registry and the Hormones and Colon Cancer study (CORE Studies).

CLUE II: We thank the participants of CLUE II and appreciate the continued efforts of the staff at the Johns Hopkins George W. Comstock Center for Public Health Research and Prevention in the conduct of the CLUE II cohort study.

COLON and NQplus: the authors would like to thank the COLON and NQplus investigators at Wageningen University and Research and the involved clinicians in the participating hospitals.

CORSA: We kindly thank all individuals who agreed to participate in the CORSA study. Furthermore, we thank all cooperating physicians and students and the Biobank Graz of the Medical University of Graz.

Cancer Prevention Study-II (CPS-II): The authors thank the CPS-II participants and Study Management Group for their invaluable contributions to this research. The authors would also like to acknowledge the contribution to this study from central cancer registries supported through the Centers for Disease Control and Prevention National Program of Cancer Registries and cancer registries supported by the National Cancer Institute Surveillance Epidemiology and End Results program.

Czech Republic CCS: We are thankful to all clinicians in major hospitals in the Czech Republic, without whom the study would not be practicable. We are also sincerely grateful to all patients participating in this study.

DACHS: We thank all participants and cooperating clinicians as well as everyone who provided excellent technical assistance.

EDRN: We acknowledge all contributors to the development of the resource at University of Pittsburgh School of Medicine, Department of Gastroenterology, Hepatology, and Nutrition; Department of Pathology; and Department of Biomedical Informatics.

EPIC: Where authors are identified as personnel of the International Agency for Research on Cancer/World Health Organization, the authors alone are responsible for the views expressed in this article, and they do not necessarily represent the decisions, policy, or views of the International Agency for Research on Cancer/World Health Organization.

EPICOLON: We are sincerely grateful to all patients participating in this study who were recruited as part of the EPICOLON project. We acknowledge the Spanish National DNA Bank, Biobank of Hospital Clínic–IDIBAPS and Biobanco Vasco for the availability of the samples. The work was carried out (in part) at the Esther Koplowitz Center, Barcelona.

Harvard cohorts (HPFS, NHS, and PHS): The study protocol was approved by the institutional review boards of the Brigham and Women’s Hospital and Harvard T.H. Chan School of Public Health and those of participating registries as required. We would like to thank the participants and staff of the HPFS, NHS, and PHS for their valuable contributions as well as the following state cancer registries for their help: Alabama, Arizona, Arkansas, California, Colorado, Connecticut, Delaware, Florida, Georgia, Idaho, Illinois, Indiana, Iowa, Kentucky, Louisiana, Maine, Maryland, Massachusetts, Michigan, Nebraska, New Hampshire, New Jersey, New York, North Carolina, North Dakota, Ohio, Oklahoma, Oregon, Pennsylvania, Rhode Island, South Carolina, Tennessee, Texas, Virginia, Washington, and Wyoming. The authors assume full responsibility for analyses and interpretation of these data.

Kentucky: We would like to acknowledge the staff at the Kentucky Cancer Registry.

Leeds Colorectal Cancer Study: We acknowledge the contributions of Gillian Smith, Emma Northwood, and Robin Waxman in conducting this study and the leadership of Jennifer Barrett, David Forman, and Roland Wolf.

NCCCS I & II: We would like to thank the study participants and the NC Colorectal Cancer Study staff.

NSHDS: The investigators thank the Västerbotten Intervention Programme, the Northern Sweden MONICA study, the Biobank Research Unit at Umeå University, and Biobanken Norr at Region Västerbotten for providing data and samples and acknowledge the contribution from Biobank Sweden, supported by the Swedish Research Council.

PLCO: The authors thank the PLCO Cancer Screening Trial screening center investigators and the staff from Information Management Services Inc and Westat Inc. Most importantly, we thank the study participants for their contributions that made this study possible. Cancer incidence data have been provided by the District of Columbia Cancer Registry, Georgia Cancer Registry, Hawaii Cancer Registry, Minnesota Cancer Surveillance System, Missouri Cancer Registry, Nevada Central Cancer Registry, Pennsylvania Cancer Registry, Texas Cancer Registry, Virginia Cancer Registry, and Wisconsin Cancer Reporting System. All are supported in part by funds from the Centers for Disease Control and Prevention, National Program for Central Registries, local states, or the National Cancer Institute’s Surveillance, Epidemiology, and End Results program. The results reported here and the conclusions derived are the sole responsibility of the authors.

SEARCH: We thank the SEARCH team.

SELECT: We thank the research and clinical staff at the sites that participated in the SELECT study, without whom the trial would not have been successful. We are also grateful to the 35,533 dedicated men who participated in SELECT.

WHI: The authors thank the WHI investigators and staff for their dedication and the study participants for making the program possible. A full listing of WHI investigators can be found at https://sp.whi.org/researchers/Documents%20%20Write%20a%20Paper/WHI%20Investigator%20Short%20List.pdf.

## Funding

This work was funded by the Genetics and Epidemiology of Colorectal Cancer Consortium (GECCO): National Cancer Institute (NCI), National Institutes of Health (NIH), and US Department of Health and Human Services (R01 CA059045, U01 CA164930, R01201407, R01CA248857, R01 CA206279, R01 CA244588, and R21 CA230486). This research was funded in part through the NIH/NCI Cancer Center Support Grant P30 CA015704. Scientific Computing Infrastructure at Fred Hutchinson Cancer Center funded by Office of Research Infrastructure Programs (ORIP) grant S10OD028685.

ASTERISK: a Hospital Clinical Research Program (PHRC-BRD09/C) from the University Hospital Center of Nantes and supported by the Regional Council of Pays de la Loire, the Groupement des Entreprises Françaises dans la Lutte contre le Cancer (GEFLUC), the Association Anne de Bretagne Génétique and the Ligue Régionale Contre le Cancer (LRCC).

The ATBC Study is supported by the Intramural Research Program of the NCI, NIH, and US Department of Health and Human Services.

BWHS is funded by NIH U01CA164974.

CLUE II funding was from the NCI (U01 CA86308, Early Detection Research Network; P30 CA006973), National Institute on Aging (U01 AG18033), and the American Institute for Cancer Research. The content of this publication does not necessarily reflect the views or policies of the US Department of Health and Human Services, nor does mention of trade names, commercial products, or organizations imply endorsement by the US government. Maryland Cancer Registry Cancer data were provided by the Maryland Cancer Registry, Center for Cancer Prevention and Control, and Maryland Department of Health, with funding from the State of Maryland and the Maryland Cigarette Restitution Fund. The collection and availability of cancer registry data are also supported by Cooperative Agreement NU58DP006333, funded by the Centers for Disease Control and Prevention. Its contents are solely the responsibility of the authors and do not necessarily represent the official views of the Centers for Disease Control and Prevention or the US Department of Health and Human Services.

ColoCare: This work was supported by the NIH (grant numbers R01 CA189184 [Christopher Li and Cornelia M. Ulrich], U01 CA206110 [Cornelia M. Ulrich, Christopher Li, Erin Siegel, Jane C. Figueiredo, and Graham Colditz], R01 CA207371 [Cornelia M. Ulrich and Christopher Li]), the Matthias LackasFoundation, the German Consortium for Translational Cancer Research, and the European Union TRANSCAN initiative.

The Colon Cancer Family Registry (CCFR) (https://www.coloncfr.org) is supported in part by funding from the NCI, NIH (award U01 CA167551). Support for case ascertainment was provided in part from the Surveillance, Epidemiology, and End Results (SEER) Program; the following US state cancer registries: Arizona, Colorado, Minnesota, North Carolina, and New Hampshire; and the Victoria Cancer Registry (Australia) and Ontario Cancer Registry (Canada). The CCFR Set-1 (Illumina 1M/1M-Duo) and Set-2 (Illumina Omni1-Quad) scans were supported by NIH awards U01 CA122839 and R01 CA143237 (to Graham Casey). The CCFR Set-3 (Affymetrix Axiom CORECT Set array) was supported by NIH award U19 CA148107 and R01 CA81488 (to Stephen B. Gruber). The CCFR Set-4 (Illumina OncoArray 600K SNP array) was supported by NIH award U19 CA148107 (to Stephen B. Gruber) and by the Center for Inherited Disease Research, which is funded by the NIH to Johns Hopkins University, contract number HHSN268201200008I. Additional funding for the OFCCR/ARCTIC was through a Cancer Risk Evaluation (CaRE) Program grant from the Canadian Cancer Society (to Steven Gallinger), and through generous support from the Ontario Ministry of Research and Innovation. The SFCCR Illumina HumanCytoSNP array was supported in part through NCI/NIH awards U01/U24 CA074794 and R01 CA076366 (to Polly A. Newcomb). The content of this manuscript does not necessarily reflect the views or policies of the NCI, NIH, or any of the collaborating centers in the Colon Cancer Family Registry (CCFR), nor does mention of trade names, commercial products, or organizations imply endorsement by the US Government, any cancer registry, or the CCFR.

COLON: The COLON study is sponsored by Wereld Kanker Onderzoek Fonds, including funds from grant 2014/1179 as part of the World Cancer Research Fund International Regular Grant Programme, by Alpe d’Huzes and the Dutch Cancer Society (UM 2012–5653, UW 2013–5927, UW2015–7946), by TRANSCAN (JTC2012-MetaboCCC, JTC2013-FOCUS) and by RegioDeal Foodvalley (grant number 162135). The Nqplus study is sponsored by a ZorgOnderzoek Nederland/Medical Sciences (ZonMW) investment grant (98–10030); by the Prevention of Diabetes Through Lifestyle Intervention and Population Studies in Europe and Around the World (PREVIEW) project, which received funding from the European Union Seventh Framework Programme (FP7/2007–2013) under grant number 312057; by funds from Top Institute Food and Nutrition (cardiovascular health theme), a public–private partnership on precompetitive research in food and nutrition; and by FOODBALL, the Food Biomarker Alliance, a project from Joint Program Initiative Healthy Diet for a Healthy Life.

COLO2&3: NIH (R01 CA60987).

Colorectal Cancer Transdisciplinary (CORECT) study: The CORECT study was supported by the NCI/NIH, US Department of Health and Human Services (grant numbers U19 CA148107, R01 CA81488, P30 CA014089, R01 CA197350, P01 CA196569, R01 CA201407, U19 CA148107, R01 CA242218) and National Institutes of Environmental Health Sciences, NIH (grant number T32 ES013678).

CORSA: The CORSA study was funded by Austrian Research Funding Agency BRIDGE (grant 829675, to Andrea Gsur) and the “Herzfelder’sche Familienstiftung” (grant to Andrea Gsur) and was supported by European Cooperation in Science and Technology (COST) Action BM1206.

Cancer Prevention Study-II (CPS-II): The American Cancer Society funds the creation, maintenance, and updating of the CPS-II cohort. This study was conducted with institutional review board approval.

Colorectal Cancer Genetics & Genomics (CRCGEN): Colorectal Cancer Genetics and Genomics, Spanish study was supported by Instituto de Salud Carlos III, cofunded by European Regional Development Fund (FEDER) funds—A Way to Build Europe (grants PI14–613 and PI09–1286), the Agency for Management of University and Research Grants (AGAUR) of the Catalan Government (grant 2017SGR723), Junta de Castilla y León (grant LE22A10–2), and Spanish Association Against Cancer Scientific Foundation grants POSTD037OBON and GCTRA18022MORE. Sample collection of this work was supported by the Xarxa de Bancs de Tumors de Catalunya sponsored by Pla Director d’Oncología de Catalunya, Plataforma Biobancos PT13/0010/0013 and ICOBIOBANC, sponsored by the Catalan Institute of Oncology.

Czech Republic CCS: This work was supported by the Grant Agency of the Czech Republic (20–03997S), by the grants AZV NV18/03/00199, and by Charles University grants Unce/Med/006 and Progress Q28/LF1.

DACHS: This work was supported by the German Research Council (BR 1704/6–1, BR 1704/6–3, BR 1704/6–4, CH 117/1–1, HO 5117/2–1, HE 5998/2–1, KL 2354/3–1, RO 2270/8–1, and BR 1704/17–1); the Interdisciplinary Research Program of the National Center for Tumor Diseases, Germany; and the German Federal Ministry of Education and Research (01KH0404, 01ER0814, 01ER0815, 01ER1505A, and 01ER1505B).

DALS: NIH (R01 CA48998 to Martha L. Slattery).

EDRN: This work is funded and supported by the NCI, EDRN grant U01 CA152753.

EPIC: The coordination of EPIC is financially supported by International Agency for Research on Cancer and also by the Department of Epidemiology and Biostatistics, School of Public Health, Imperial College London, which has additional infrastructure support provided by the National Institute for Health and Care Research (NIHR) Imperial Biomedical Research Center. The national cohorts are supported by the Danish Cancer Society (Denmark); Ligue Contre le Cancer, Institut Gustave Roussy, Mutuelle Générale de l’Education Nationale, Institut National de la Santé et de la Recherche Médicale (INSERM) (France); German Cancer Aid, German Cancer Research Center (DKFZ), German Institute of Human Nutrition Potsdam-Rehbruecke, Federal Ministry of Education and Research (Germany); Associazione Italiana per la Ricerca sul Cancro-AIRC-Italy, Compagnia di SanPaolo, and National Research Council (Italy); Dutch Ministry of Public Health, Welfare, and Sports; Netherlands Cancer Registry; LK Research Funds; Dutch Prevention Funds; Dutch ZON (Zorg Onderzoek Nederland); World Cancer Research Fund; and Statistics Netherlands (Netherlands); Health Research Fund–Instituto de Salud Carlos III; Regional Governments of Andalucía, Asturias, Basque Country, Murcia, and Navarra; and the Catalan Institute of Oncology (ICO), Spain; Swedish Cancer Society, Swedish Research Council, Region Skåne, and Region Västerbotten (Sweden); Cancer Research UK (14136 to EPIC-Norfolk; C8221/A29017 to EPIC-Oxford) and Medical Research Council (1000143 to EPIC-Norfolk; MR/M012190/1 to EPIC-Oxford) (United Kingdom).

EPICOLON: This work was supported by grants from Fondo de Investigación Sanitaria/FEDER (PI08/0024, PI08/1276, PS09/02368, P111/00219, PI11/ 00681, PI14/00173, PI14/00230, PI17/00509, 17/00878, PI20/00113, PI20/00226, Acción Transversal de Cáncer), Xunta de Galicia (PGIDIT07PXIB9101209PR), Ministerio de Economia y Competitividad (SAF07–64873, SAF 2010–19273, SAF2014–54453R), Fundación Científica de la Asociación Española contra el Cáncer (GCB13131592CAST), Beca Grupo de Trabajo “Oncología” (Asociación Española de Gastroenterología), Fundación Privada Olga Torres, FP7 CHIBCHA Consortium, Agència de Gestió d’Ajuts Universitaris i de Recerca (Generalitat de Catalunya, 2014SGR135, 2014SGR255, 2017SGR21, 2017SGR653), Catalan Tumour Bank Network (Pla Director d’Oncologia, Generalitat de Catalunya), PERIS (SLT002/16/00398, Generalitat de Catalunya), CERCA Programme (Generalitat de Catalunya), and COST Actions BM1206 and CA17118. Centro de Investigación Biomédica en Red de Enfermedades Hepáticas y Digestivas is funded by the Instituto de Salud Carlos III.

ESTHER/VERDI: This work was supported by grants from the Baden-Württemberg Ministry of Science, Research, and Arts and the German Cancer Aid.

Harvard cohorts (HPFS, NHS, PHS): HPFS is supported by the NIH (P01 CA055075, UM1 CA167552, U01 CA167552, R01 CA137178, R01 CA151993, and R35 CA197735), NHS is supported by the NIH (R01 CA137178, P01 CA087969, UM1 CA186107, R01 CA151993, and R35 CA197735) and PHS is supported by the NIH (R01 CA042182).

Hawaii Adenoma Study: NCI grant R01 CA072520.

HCCS: We acknowledge funding support for this project from the NIH (R01CA155101, R01CA238087, and P20CA252733).

Kentucky: This work was supported by the following grant support: Clinical Investigator Award from Damon Runyon Cancer Research Foundation (CI-8), NCI R01CA136726.

LCCS: The LCCS study was funded by the Food Standards Agency and Cancer Research UK Programme Award (C588/A19167).

MCCS cohort recruitment was funded by VicHealth and Cancer Council Victoria. The MCCS was further supported by Australian National Health and Medical Research Council (NHMRC) grants 509348, 209057, 251553, and 504711 and by infrastructure provided by Cancer Council Victoria. Cases and their vital status were ascertained through the Victorian Cancer Registry and the Australian Institute of Health and Welfare, including the National Death Index and the Australian Cancer Database.

MEC: NIH (R37 CA054281, P01 CA033619, U01 CA14973, and R01 CA063464).

MECC: This work was supported by the NIH, US Department of Health and Human Services (R01 CA081488, R01 CA197350,U19 CA148107, R01 CA242218).

Memorial Sloan Kettering Cancer Center: The work at Sloan Kettering in New York was supported by the Robert and Kate Niehaus Center for Inherited Cancer Genomics and the Romeo Milio Foundation.

Moffitt: This work was supported by funding from the NIH (grant numbers R01 CA189184, P30 CA076292), Florida Department of Health Bankhead-Coley Grant 09BN-13, and the University of South Florida Oehler Foundation. Moffitt contributions were supported in part by the Total Cancer Care Initiative, Collaborative Data Services Core, and Tissue Core at the H. Lee Moffitt Cancer Center & Research Institute, and NCI-designated Comprehensive Cancer Center (grant number P30 CA076292).

NCCCS I & II: We acknowledge funding support for this project from the NIH (R01 CA66635 and P30 DK034987).

NFCCR: This work was supported by an Interdisciplinary Health Research Team award from the Canadian Institutes of Health Research (CRT 43821); the NIH, US Department of Health and Human Services (U01 CA74783); and National Cancer Institute of Canada grants (18223 and 18226). The authors wish to acknowledge the contribution of Alexandre Belisle and the genotyping team of the McGill University and Génome Québec Innovation Center, Montréal, Canada, for genotyping the Sequenom panel in the NFCCR samples.

NSHDS: The research was supported by Biobank Sweden through funding from the Swedish Research Council (VR 2017–00650, VR 2017–01737); the Swedish Cancer Society (CAN 2017/581); Region Västerbotten (VLL-841671, VLL-833291); the Knut and Alice Wallenberg Foundation (VLL-765961); and the Lion’s Cancer Research Foundation (several grants) and Insamlingsstiftelsen, both at Umeå University.

OSUMC: The data reported here were derived from the Ohio Colorectal Cancer Prevention Initiative (OCCPI), supported by a grant from Pelotonia, an annual cycling event in Columbus, Ohio, that supports cancer research at The Ohio State University Comprehensive Cancer Center—James Cancer Hospital and Solove Research Institute. OCCPI was also supported in part by grant number P30 CA016058, NCI, Bethesda, Maryland, and utilized the Biospecimen Services Shared Resource Biorepository of the Ohio State University Comprehensive Cancer Center. Myriad Genetics Laboratories donated germline next-generation sequencing testing for selected mismatch repair-proficient patients. HNPCC funding was provided by the NCI (CA016058 and CA067941).

PLCO: Intramural Research Program of the Division of Cancer Epidemiology and Genetics and supported by contracts from the Division of Cancer Prevention, NCI, NIH, US Department of Health and Human Services. Funding was provided by the NIH’s Genes, Environment, and Health Initiative (Z01 CP 010200, NIH U01 HG004446, and NIH GEI U01 HG 004438).

SEARCH: The University of Cambridge has received salary support in respect of Pharoah PDP from the NHS in the East of England through the Clinical Academic Reserve; Cancer Research UK (C490/A16561); and the UK National Institute for Health Research Biomedical Research Centers at the University of Cambridge.

SMS: This work was supported by the NCI (grant P01 CA074184 to John D. Potter and Polly A. Newcombe; grants R01 CA097325, R03 CA153323, and K05 CA152715 to Polly A. Newcombe; and the National Center for Advancing Translational Sciences at the NIH (grant KL2 TR000421 to Andrea N. Burnett-Hartmann).

The Swedish Low-Risk Colorectal Cancer Study: The study was supported by grants from the Swedish research council (K2015–55X-22674–01-4, K200855X-20157–03-3, K2006–72X-20157–01-2) and the Stockholm County Council (ALF project).

Swedish Mammography Cohort and Cohort of Swedish Men: This work is supported by the Swedish Research Council/Infrastructure grant, the Swedish Cancer Foundation, and the Karolinska Institute’s Distinguished Professor Award to Alicja Wolk.

UK Biobank: This research has been conducted using the UK Biobank Resource under application number 8614.

VITAL: NIH (K05 CA154337).

WHI: The WHI program is funded by the National Heart, Lung, and Blood Institute, NIH, US Department of Health and Human Services through contracts HHSN268201100046C, HHSN268201100001C, HHSN268201100002C, HHSN268201100003C, HHSN268201100004C, and HHSN271201100004C.

Daniel D. Buchanan is supported by an NHMRC Emerging Leadership Investigator grant (GNT1194896), University of Melbourne Dame Kate Campbell Fellowship, and funding from the University of Melbourne Research at Melbourne Accelerator Program. Peter Georgeson is supported by an Australian Government Research Training Program Scholarship.
