# Supplementary material for: Elucidating the Risk of Colorectal Cancer for Variants in Hereditary Colorectal Cancer Genes
Source: Gastroenterology. Author manuscript; Available in PMC 2024 Feb 14. (PMC10866455; doi:10.1053/j.gastro.2023.06.032)
Supplement: 1 [file NIHMS1962932-supplement-1.pdf]

Supplementary Table 1. Characteristics of studies and study participants contributing to the GWAS meta-analysis.

						Cases			Controls		
**Study Acronym**	**Study Name**	**Study Design**	**Country**	**Genotyping Platform**	**N total**	**N Cases (Ad)**	**N Females (%)**	**Mean age (SD), years**	**N Controls**	**N Females (%)**	**Mean age (SD), years**
ASTERISK	Association STudy Evaluating RISK for sporadic colorectal cancer	case-control	France	Illumina 300K	1838	892(0)	340(38.1)	F: 69.3 (10.8) M: 68.4(9.4)	946	423(44.7)	F: 62.7 (10.5) M: 61.3(9.8)
ATBC	Alpha-Tocopherol, Beta Carotene Cancer Prevention Study	cohort	Finland	Illumina Oncoarray	184	152(0)	0(0)	F: 0 (0) M: 57.5(4.9)	32	0(0)	F: 0 (0) M: 57.1(4.3)
CCFR_1	Colon Cancer Family Registry	case-control	USA, Canada, Australia	Illumina 1M, 1M duo	1989	1020(0)	497(48.7)	F: 50.1 (11.6) M: 52.8(11.3)	969	503(51.9)	F: 57.5 (11.3) M: 58.5(10.4)
CCFR_2	Colon Cancer Family Registry	case-control	USA, Canada, Australia	Illumina 1M, 1M duo	635	322(0)	168(52.2)	F: 49.2 (10.1) M: 52.2(11.1)	313	167(53.4)	F: 52.6 (10.3) M: 53(11.8)
CCFR_3	Colon Cancer Family Registry	case-control	USA, Canada, Australia	Affymetrix Axiom	2655	1588(0)	871(54.8)	F: 56.7 (11.2) M: 54.3(11.1)	1067	539(50.5)	F: 55.3 (11.9) M: 56.6(11.1)
CCFR_4	Colon Cancer Family Registry	case-control	USA, Canada, Australia	Illumina Oncoarray	2525	1873(0)	915(48.9)	F: 53.5 (12.1) M: 54.3(11.1)	652	302(46.3)	F: 49.5 (13.2) M: 53.2(13.2)
CGN	USC Norris Comprehensive Cancer Center Genetics Registry	case-series	USA	Oncoarray	157	157(0)	72(45.9)	F: 47.8 (14.4) M: 49(9.2)			
CLUEII	Campaign against Cancer and Heart Disease II	cohort	USA	Illumina Oncoarray+custom iSelect	517	265(0)	141(53.2)	F: 61.8 (11.9) M: 61.2(10.7)	252	135(53.6)	F: 61.7 (12.1) M: 60.3(10.7)
Colo2&3	Hawai'i Colorectal Cancer Studies 2&3	case-series	USA	Illumina 300K	211	87(0)	40(46)	F: 66.3 (12) M: 65.2(11.5)	124	54(43.5)	F: 65.6 (10.5) M: 64.5(11.4)
ColoCare_Heidelberg	ColoCare Consortium	case-series	Germany	Illumina Oncoarray	148	110(0)	37(33.6)	F: 61.8 (11.5) M: 59.4(12.2)	38	21(55.3)	F: 52.1 (11.4) M: 59.4(14.5)
ColoCare_Seattle	ColoCare Consortium	case-series	USA	Illumina Oncoarray	169	169(0)	73(43.2)	F: 56.1 (12.1) M: 57.2(13.5)	0	0(0)	F:0 (0) M: 0(0)
COLON	Colorectal Cancer: Longitudinal Observational study on Nutritional and lifestyle factors that influence colorectal tumor recurrence, survival and quality of life	case-control	Netherlands	Illumina OmniExpressExome	1337	640(0)	235(36.7)	F: 64.4 (10.1) M: 65.9(8.9)	697	259(37.2)	F: 60.8 (6.9) M: 62.1(6.2)
CORSA_1	Colorectal Cancer Study of Austria	case-control	Austria	Affymetrix Axiom	2183	1459(525)	549(37.6)	F: 65.1 (11.2) M: 64(10.8)	724	314(43.4)	F: 63 (11.1) M: 63.1(10)
CORSA_2	Colorectal Cancer Study of Austria	case-control	Austria	Illumina Oncoarray+custom iSelect	2470	1245(712)	439(35.3)	F: 65.8 (11.1) M: 65.5(10.6)	1225	439(35.8)	F: 60.8 (12.3) M: 56(13)
CPSII_1	American Cancer Society Cancer Prevention Study II nested case-control study	cohort	USA	Affymetrix Axiom	1078	542(0)	270(49.8)	F: 68.5 (5.7) M: 68.9(5.3)	536	259(48.3)	F: 68.1 (5.7) M: 69(5.4)
CPSII_2	American Cancer Society Cancer Prevention Study II nested case-control study	cohort	USA	Illumina Oncoarray+custom iSelect	687	340(0)	177(52.1)	F: 68.3 (6.1) M: 69.2(5.2)	347	182(52.4)	F: 68 (6.1) M: 69.2(5.2)
CRCGEN	Colorectal Cancer Genetics & Genomics, Spanish study	case-control	Spain	Illumina Oncoarray	1907	885(0)	318(35.9)	F: 67 (11.7) M: 67.9(10.3)	1022	495(48.4)	F: 62.6 (11.8) M: 66.1(10.3)
Czech	Czech Republic CCS	case-control	Czech Republic	Illumina Oncoarray+custom iSelect	3297	1681(0)	630(37.5)	F: 62.9 (11.8) M: 63.7(10.8)	1616	734(45.4)	F: 50.2 (14.7) M: 50.9(12.5)
DACHS_1	Darmkrebs: Chancen der Verhütung durch Screening	case-control	Germany	Illumina 300K	3327	1650(0)	690(41.8)	F: 69.2 (11.2) M: 67.8(9.7)	1677	675(40.3)	F: 69.1 (10) M: 68.7(10.2)
DACHS_2	Darmkrebs: Chancen der Verhütung durch Screening	case-control	Germany	Illumina OmniExpress	1142	651(0)	256(39.3)	F: 69.7 (11.9) M: 68.3(10.4)	491	174(35.4)	F: 70.8 (10.3) M: 69(9.4)
DACHS_3	Darmkrebs: Chancen der Verhütung durch Screening Study	case-control	Germany	Illumina OmniExpressExome	1837	1217(0)	459(37.7)	F: 69.6 (11.3) M: 68.1(10.4)	620	245(39.5)	F: 65.8 (12.8) M: 67.3(10.6)
DALS_1	Diet, Activity and Lifestyle Study	case-control	USA	Illumina 550K, 610K	1402	700(0)	303(43.3)	F: 64.5 (9.9) M: 63.3(9.4)	702	306(43.6)	F: 64.3 (9.9) M: 63.2(9.9)
DALS_2	Diet, Activity and Lifestyle Study	case-control	USA	Illumina 300K	860	399(0)	189(47.4)	F: 63.5 (10.4) M: 64.2(9.9)	461	220(47.7)	F: 64.3 (9.8) M: 64.3(10.2)
EDRN	Early Detection Research Network	case cohort	USA	Illumina Oncoarray+custom iSelect	590	278(6)	109(39.2)	F: 62.4 (13.3) M: 62.7(11.6)	312	160(51.3)	F: 59 (10.4) M: 60(10.7)
EPIC	European Prospective Investigation into Cancer and Nutrition	cohort	Europe	Illumina OmniExpressExome	4339	2022(0)	1084(53.6)	F: 57 (8.2) M: 57.2(8.2)	2317	1239(53.5)	F: 56.6 (8) M: 56.8(8.1)
EPICOLON	EPICOLON	case-control	Spain	Illumina Oncoarray+custom iSelect	609	268(0)	111(41.4)	F: 54.3 (10.3) M: 57.6(9.1)	341	142(41.6)	F: 58.7 (6.6) M: 59.8(6.4)
ESTHER_VERDI	Epidemiologische Studie zu Chancen der Verhütung, Früherkennung und optimierten Therapie chronischer Erkrankungen in der älteren Bevölkerung; Verlauf der diagnostischen Abklärung bei Krebspatienten	case-control	Germany	Illumina Oncoarray	853	417(0)	143(34.3)	F: 65.2 (8.5) M: 64.8(6.6)	436	153(35.1)	F: 65.1 (7.2) M: 64.9(5.9)
FIRE3	5-FU, Folinic Acid and Irinotecan (FOLFIRI) Plus Cetuximab Versus FOLFIRI Plus	case-series	Germany, Austria	Oncoarray	232	232(0)	67(28.9)	F: 62.3 (8.8) M: 62.3(8.7)			
GALEON	GALicia Estudio Oncológico de coloN	case-series	USA,Spain	Oncoarray	92	92(0)	37(40.2)	F: 68.6 (10) M: 70.7(9.5)			
HawaiiCCS_AD	Hawaii Adenoma Study	case-control	USA	Illumina Oncoarray+custom iSelect	610	80(80)	32(40)	F: 60.2 (9.4) M: 59(8.1)	530	191(36)	F: 61.9 (8.8) M: 60.5(7.7)
HispanicCCS	Hispanic Colorectal Cancer Study	case-series	USA	Oncoarray	4	4(0)	1(25)	F: 31 (0) M: 66.7(11.9)			
HPFS_1	Health Professionals Follow-Up Study	cohort	USA	Illumina OmniExpress	449	221(0)	0(0)	F: 0 (0) M: 66.6(8.6)	228	0(0)	F: 0 (0) M: 66.1(8.9)
HPFS_2	Health Professionals Follow-Up Study	cohort	USA	Illumina OmniExpress	343	173(0)	0(0)	F: 0 (0) M: 63.4(8.5)	170	0(0)	F: 0 (0) M: 64(8.9)
HPFS_3_AD	Health Professionals Follow-Up Study	cohort	USA	Illumina OmniExpress	597	309(309)	0(0)	F: 0 (0) M: 60.9(8.6)	288	0(0)	F: 0 (0) M: 60.3(8.4)
HPFS_4	Health Professionals Follow-Up Study	cohort	USA	Illumina OmniExpressExome	381	183(0)	0(0)	F: 0 (0) M: 71.3(8.7)	198	0(0)	F: 0 (0) M: 70.8(8.8)
HPFS_5_AD	Health Professionals Follow-Up Study	cohort	USA	Illumina Oncoarray+custom iSelect	260	155(155)	0(0)	F: 0 (0) M: 66.1(7.9)	105	0(0)	F: 0 (0) M: 66.6(7.9)
Kentucky	Kentucky Case-Control Study	case-control	USA	Affymetrix Axiom	2093	961(0)	487(50.7)	F: 61.6 (10.3) M: 62.1(10.1)	1132	572(50.5)	F: 66.7 (6.6) M: 60.4(9.2)
LCCS	Leeds Colorectal Cancer Study	case-control	UK	Illumina Oncoarray+custom iSelect	2092	1409(0)	568(40.3)	F: 70.3 (11.1) M: 69.1(9.8)	683	362(53)	F: 67.7 (9) M: 68.5(7.8)
MAVERICC	MAVERICC	case-series	USA	Oncoarray	232	232(0)	85(36.6)	F: 58.2 (11.1) M: 62.8(10.1)			
MCCS_1	Melbourne Collaborative Cohort Study	cohort	Australia	Affymetrix Axiom	995	534(0)	257(48.1)	F: 59.9 (7.7) M: 59.6(7.4)	461	226(49)	F: 59.5 (7.2) M: 59.6(8.1)
MCCS_2	Melbourne Collaborative Cohort Study	cohort	Australia	Affymetrix Axiom	430	218(0)	104(47.7)	F: 58.2 (8.2) M: 58.6(7)	212	99(46.7)	F: 60.1 (7.5) M: 60.7(7.3)
MEC_1	Multiethnic Cohort Study	cohort	USA	Illumina 300K	661	321(0)	144(44.9)	F: 63.5 (7.5) M: 62.6(8.4)	340	159(46.8)	F: 63.3 (7.7) M: 62.8(8.3)
MEC_2	Multiethnic Cohort Study	cohort	USA	Illumina 300K	155	66(0)	35(53)	F: 60.3 (8.8) M: 59.8(9.2)	89	43(48.3)	F: 64.3 (8.1) M: 62.1(8.9)
MECC_1	Molecular Epidemiology of Colorectal Cancer Study	case-control	Israel	Illumina OmniExpress	952	474(0)	215(45.4)	F: 71 (10.9) M: 73(9.2)	478	224(46.9)	F: 71.3 (10.6) M: 73.3(9)
MECC_2	Molecular Epidemiology of Colorectal Cancer Study	case-control	Israel	Affymetrix Axiom	2167	1112(0)	559(50.3)	F: 71.1 (11) M: 72.3(10.5)	1055	534(50.6)	F: 71.4 (11.5) M: 73.4(10.7)
MECC_3	Molecular Epidemiology of Colorectal Cancer Study	case-control	Israel	Illumina Oncoarray	6396	3600(0)	1704(47.3)	F: 67.5 (13) M: 68.8(12.2)	2796	1316(47.1)	F: 69.4 (12.9) M: 70.4(11.8)
MOFFITT	Colorectal Cancer Outcomes Prognosis and Epidemiology (COPE) Study and Total	case-series	USA	Oncoarray	379	379(0)	172(45.4)	F: 68.7 (10.7) M: 67.1(10)			
MSKCC	Memorial Sloan Kettering Cancer Center Cohort	case-control	USA	Illumina Oncoarray	77	77(0)	47(61)	F: 57.9 (14) M: 61.1(12.3)			
NCCCSI	North Carolina Colon Cancer Study, I	case-control	USA	Illumina Oncoarray+custom iSelect	706	242(0)	106(43.8)	F: 65.2 (10) M: 65(9.5)	464	204(44)	F: 66.1 (9.5) M: 66.1(9.6)
NCCCSII	North Carolina Colon Cancer Study, II	case-control	USA	Illumina Oncoarray+custom iSelect	1281	595(0)	241(40.5)	F: 62.3 (10.4) M: 62.2(10.4)	686	258(37.6)	F: 63.4 (10.7) M: 64.6(9.1)
NFCCR_2	Newfoundland Case-Control Study	case-control	Canada	Affymetrix Axiom	664	193(0)	73(37.8)	F: 60.1 (8.6) M: 62.7(8.4)	471	194(41.2)	F: 58.1 (8.5) M: 60.1(9.3)
NGCCS	PopGen Biobank	case-control	Germany	Illumina Oncoarray	1115	1115(0)	488(43.8)	F: 58.8 (9.6) M: 61(8.4)			
NHS_1	Nurses' Health Study	cohort	USA	Illumina OmniExpress	1163	389(0)	389(100)	F: 60 (6.6) M: 0(0)	774	774(100)	F: 60 (6.5) M: 0(0)
NHS_2	Nurses' Health Study	cohort	USA	Illumina OmniExpress	338	157(0)	157(100)	F: 58.5 (6.4) M: 0(0)	181	181(100)	F: 59.5 (6.3) M: 0(0)
NHS_3_AD	Nurses' Health Study	cohort	USA	Illumina OmniExpress	916	500(500)	500(100)	F: 56.8 (6.7) M: 0(0)	416	416(100)	F: 56.1 (6.6) M: 0(0)
NHS_4	Nurses' Health Study	cohort	USA	Illumina OmniExpressExome	599	298(1)	298(100)	F: 69.4 (9) M: 0(0)	301	301(100)	F: 69.4 (8.7) M: 0(0)
NHS_5_AD	Nurses' Health Study	cohort	USA	Illumina Oncoarray+custom iSelect	465	247(247)	247(100)	F: 66.1 (7.6) M: 0(0)	218	218(100)	F: 66.6 (7.8) M: 0(0)
NHSII	Nurses' Health Study	cohort	USA	Illumina Oncoarray	210	109(0)	109(100)	F: 36.5 (4.5) M: 0(0)	101	101(100)	F: 36.3 (4.2) M: 0(0)
NSHDS	The Northern Sweden Health and Disease Study	cohort	Sweden	Illumina Oncoarray+custom iSelect	823	409(0)	262(64.1)	F: 57.4 (7.7) M: 53.3(7.3)	414	261(63)	F: 57.7 (7.7) M: 53.4(7.3)
OSUMC	Columbus-area HNPCC study, Ohio Colorectal Cancer Prevention Initiative, Ohio State University Medical Center	case-control	USA	Illumina Oncoarray+custom iSelect	5545	3109(0)	1432(46.1)	F: 62.3 (13.2) M: 61.4(12.9)	2436	1476(60.6)	F: 51.4 (15) M: 53.4(14.4)
PHS	Physicians' Health Study	cohort	USA	Illumina OmniExpress	761	374(0)	0(0)	F: 0 (0) M: 59.3(9.1)	387	0(0)	F: 0 (0) M: 58.6(8.9)
PLCO_1	Prostate, Lung, Colorectal, and Ovarian Cancer Screening Trial	cohort	USA	Illumina 300/240S & 610K	2068	678(135)	251(37)	F: 64.6 (5.3) M: 64.6(5)	1390	322(23.2)	F: 63.3 (5) M: 64(5.1)
PLCO_2	Prostate, Lung, Colorectal, and Ovarian Cancer Screening Trial	cohort	USA	Illumina 300K	837	470(5)	198(42.1)	F: 63.7 (5.4) M: 63.7(5.2)	367	163(44.4)	F: 63.6 (5.1) M: 63.5(5.3)
PLCO_3	Prostate, Lung, Colorectal, and Ovarian Cancer Screening Trial	cohort	USA	Oncoarray	4135	739(568)	299(40.5)	F: 63.2 (5.2) M: 62.6(5.3)	3396	1975(58.2)	F: 62.1 (5.3) M: 61.9(5.3)
PLCO_4_AD	Prostate, Lung, Colorectal, and Ovarian Cancer Screening Trial	cohort	USA	Illumina Oncoarray+custom iSelect	2032	1102(1097)	437(39.7)	F: 62.8 (5.4) M: 62.3(5.2)	930	592(63.7)	F: 62 (5.3) M: 62(5.3)
PMH-CCFR	Postmenopausal Hormones Supplementary Study to the Colon Cancer Family Registry	case-control	USA	Illumina 300K	128	10(0)	10(100)	F: 62.2 (8) M: 0(0)	118	118(100)	F: 61.8 (7.4) M: 0(0)
PPS3	Aspirin/Folate Polyp Prevention Study	case-control	USA	Oncoarray	520	68(60)	17(25)	F: 57.5 (8.5) M: 61.3(8.6)	452	186(41.2)	F: 55.6 (9.9) M: 56.7(9)
PPS4	Vitamin D/Calcium Polyp Prevention Study	case-control	USA	Oncoarray	1053	143(135)	33(23.1)	F: 58.5 (7.8) M: 59.9(6.9)	910	377(41.4)	F: 56.3 (6.1) M: 58.2(6.6)
PURIFICAR	Puerto Rico Familial Colorectal Cancer Registry	case-control	USA	Oncoarray	156	82(0)	46(56.1)	F: 59.3 (11.3) M: 63.7(10.5)	74	48(64.9)	F: 53.5 (10.9) M: 58.5(12.5)
SEARCH	Studies of Epidemiology and Risk Factors in Cancer Heredity	case-control	UK	Illumina Oncoarray	5375	4226(0)	1805(42.7)	F: 62.7 (8.2) M: 63.8(7.4)	1149	1016(88.4)	F: 36.5 (15.7) M: 54.8(7.6)

SELECT	Selenium and Vitamin E Prevention Trial	clinical trial	USA	Illumina Oncoarray+custom iSelect	530	262(0)	0(0)	F: 0 (0) M: 65.9(6.3)	268	0(0)	F: 0 (0) M: 65.8(6.6)
SLRCCS	Swedish Low-Risk Colorectal Cancer Study	cohort	Sweden	Illumina Oncoarray	5146	2653(0)	1194(45)	F: 68.2 (11.1) M: 68.5(10.5)	2493	1177(47.2)	F: 58.3 (15.7) M: 59.6(15.3)
SMC_COSM	Swedish Mammography Cohort and Cohort of Swedish Men	cohort	Sweden	Illumina Oncoarray	1434	578(0)	238(41.2)	F: 63.9 (7.8) M: 63.7(8.1)	856	366(42.8)	F: 63.7 (8.2) M: 63.7(8.1)
SMS_AD	Screening Markers for Colorectal Cancer Study (advanced adenomas)	case-control	USA	Illumina Oncoarray+custom iSelect	168	41(41)	16(39)	F: 65.6 (10.7) M: 63.4(9.3)	127	71(55.9)	F: 58.7 (11.2) M: 63.2(11)
TRIBE	Combination Chemotherapy and Bevacizumab as First-Line Therapy in Treating	case-control	Italy	Oncoarray	321	321(0)	125(38.9)	F: 58 (9.2) M: 59.4(9.5)			
UKB	UK Biobank	cohort	UK	UK Biobank Axiom	26745	5377(354)	2282(42.4)	F: 61.2 (6.3) M: 62.1(5.8)	21368	9064(42.4)	F: 59.3 (7.2) M: 60.4(6.7)
USC_HRT_CRC	Los Angeles County Cancer Surveillance Program	case-control	USA	Illumina Oncoarray	697	297(0)	297(100)	F: 65.3 (5.5) M: 0(0)	400	400(100)	F: 63.9 (6.8) M: 0(0)
VITAL	VITamins And Lifestyle	cohort	USA	Illumina 300K	552	270(0)	124(45.9)	F: 66.6 (6) M: 65.9(6.3)	282	136(48.2)	F: 67.3 (6.1) M: 66(6.4)
WHI_1	Women's Health Initiative Study	cohort	USA	Illumina 550K, 550Kduo, 610K	1981	471(0)	471(100)	F: 67.4 (6.8) M: 0(0)	1510	1510(100)	F: 69.5 (6.4) M: 0(0)
WHI_2	Women's Health Initiative Study	cohort	USA	Illumina 300K	1969	978(0)	978(100)	F: 66 (6.6) M: 0(0)	991	991(100)	F: 65.6 (6.2) M: 0(0)
WHI_3	Women's Health Initiative Study	cohort	USA	Illumina Oncoarray+custom iSelect	1115	556(0)	556(100)	F: 65.1 (6.5) M: 0(0)	559	559(100)	F: 64.4 (6.3) M: 0(0)

Supplementary Table 2. List of variants with p-value<0.05 in CRC genes with ClinVar classifications benign (B), likely benign (LB), VUS or pathogenic (P). *p-value<0.01

Gene	Inheritance category	Variant	Exon	gnomAD AF	MAF in study	p-value (*<0.01)	ClinVar
MUTYH	AR	rs36053993	13	3.34E-03	5.74E-03	2.17E-05*	P
MUTYH	AR	rs587780741	12	2.79E-05	4.48E-06	1.46E-02	VUS
MUTYH	AR	rs34612342	7	1.64E-03	2.09E-03	3.53E-03*	P
MUTYH	AR	rs201678305	-	5.30E-03	2.71E-05	7.10E-03*	None
EPCAM		rs900435503	1	None	0	5.01E-03*	None
EPCAM		rs545339093	1	8.16E-04	1.03E-03	3.66E-02	LB
EPCAM		rs772303281	2	None	7.00E-05	3.89E-02	None
EPCAM		rs189732445	4	2.23E-04	7.90E-04	3.80E-02	B
MSH2	AD	rs374135434	7	2.09E-05	1.25E-05	4.08E-02	VUS
MSH2	AD	rs63750379	7	6.98E-06	6.33E-06	3.63E-02	VUS
MSH2	AD	rs587778524	10	None	3.83E-06	1.66E-02	VUS
MSH2	AD	rs774440277	14	2.09E-05	1.56E-05	2.13E-02	VUS
MSH6	AD	rs201132087	1	1.40E-05	0	4.89E-02	None
MSH6	AD	rs1392893164	-	7.07E-05	8.00E-05	9.43E-03*	None
MSH6	AD	rs146469162	3	3.28E-04	2.00E-05	3.00E-02	VUS
MSH6	AD	rs1800938	4	1.05E-04	9.00E-05	2.80E-02	LB
MSH6	AD	rs1336187952	4	None	6.00E-05	1.29E-02	VUS
MSH6	AD	rs200938360	4	4.19E-05	1.00E-04	3.98E-03*	VUS
MSH6	AD	rs752435825	4	6.98E-06	4.00E-05	2.55E-02	VUS
MSH6	AD	rs373721483	4	4.88E-05	0	3.84E-02	VUS
MSH6	AD	rs63751450	4	4.19E-05	1.70E-04	3.33E-02	VUS
MSH6	AD	rs730881801	4	3.49E-05	3.00E-05	1.55E-02	VUS
MSH6	AD	rs1461013021	5	None	0	2.43E-02	None
MSH6	AD	rs369583604	6	2.09E-05	0	4.70E-02	VUS
MSH6	AD	rs63750554	8	2.79E-05	6.20E-04	3.43E-02	VUS
MSH6	AD	rs863224627	10	7.11E-06	7.00E-05	4.61E-03*	VUS

MSH6	AD	rs1209834047	10	None	0	6.63E-03*	VUS
MLH1	AD	rs41295280	1	1.60E-04	2.03E-04	1.29E-02	VUS
MLH1	AD	rs758040210	6	2.09E-05	4.84E-07	2.53E-02	VUS
MLH1	AD	rs150478207	8	1.19E-04	3.63E-05	4.04E-02	VUS
MLH1	AD	rs2308317	8	8.01E-03	1.13E-04	1.87E-02	B
MLH1	AD	rs63750365	12	3.28E-04	7.18E-04	5.50E-03*	B
MLH1	AD	rs138584384	19	6.98E-06	1.64E-05	1.87E-02	VUS
MLH1	AD	rs566928243	19	5.58E-05	6.59E-04	1.96E-02	VUS
MSH3	AD	rs149350323	2	1.26E-04	9.00E-05	1.57E-02	None
MSH3	AD	rs202184623	5	3.49E-05	2.00E-05	3.36E-02	None
MSH3	AD	rs1418423507	8	6.98E-06	2.00E-05	3.25E-02	None
MSH3	AD	rs766948921	9	2.79E-05	8.00E-05	1.93E-02	None
MSH3	AD	rs749116282	10	1.40E-05	5.00E-05	4.33E-02	None
MSH3	AD	rs55724159	11	2.15E-03	2.30E-04	4.12E-02	None
MSH3	AD	rs764832633	13	1.40E-05	1.00E-05	4.48E-02	None
MSH3	AD	rs35045151	14	8.66E-04	8.00E-05	4.61E-02	None
MSH3	AD	rs200612739	15	3.49E-05	2.00E-05	3.08E-02	None
MSH3	AD	rs148896355	15	1.26E-04	0	3.11E-02	None
MSH3	AD	rs200819607	16	2.72E-04	3.60E-04	6.84E-04*	None
MSH3	AD	rs10067975	17	2.09E-05	5.00E-05	4.28E-02	None
APC	AD	rs200089324	4	2.79E-05	3.95E-05	4.38E-02	VUS
APC	AD	rs185154886	6	7.68E-05	1.67E-05	4.23E-02	None
APC	AD	rs76685252	7	2.80E-05	3.54E-06	4.87E-02	VUS
APC	AD	rs587783034	15	2.09E-05	3.29E-07	3.29E-02	VUS
APC	AD	rs33974176	17	8.98E-03	7.08E-05	2.09E-02	B
APC	AD	rs147394539	17	3.84E-04	8.16E-06	9.69E-03*	VUS
APC	AD	rs202168805	17	4.19E-05	9.07E-05	4.26E-02	VUS
APC	AD	rs1801155	17	1.15E-03	5.42E-03	1.62E-14*	VUS
APC	AD	rs587780597	17	1.40E-05	1.02E-05	1.60E-02	VUS
APC	AD	rs730881253	17	1.40E-05	6.67E-05	1.35E-02	VUS
APC	AD	rs1269798265	17	1.40E-05	2.15E-05	1.87E-02	None

APC	AD	rs141010008	17	3.00E-04	5.40E-04	4.10E-02	VUS
APC	AD	rs145444830	17	6.98E-05	1.13E-04	3.64E-02	VUS
APC	AD	rs371616945	17	2.79E-05	2.27E-05	8.11E-03*	VUS
APC	AD	rs780049836	17	2.02E-04	1.74E-06	2.68E-02	VUS
PMS2	AD	rs199700509	11	3.49E-05	4.31E-05	1.95E-02	VUS
PMS2	AD	rs536111818	11	4.19E-05	9.80E-05	2.01E-02	VUS
PMS2	AD	rs768480216	11	1.40E-05	5.81E-07	3.72E-02	VUS
PMS2	AD	rs1173245928	7	7.08E-06	8.95E-09	1.17E-02	None
PMS2	AD	rs773752935	6	None	5.70E-06	4.83E-02	None
PMS2	AD	rs786204133	5	2.09E-05	6.06E-05	2.16E-02	VUS
PMS2	AD	rs1057521802	3	6.98E-06	9.15E-05	3.90E-02	VUS
PMS2	AD	rs148270248	2	4.19E-05	5.60E-06	2.12E-02	VUS
PMS2	AD	rs201343342	2	1.54E-04	6.97E-04	2.29E-02	VUS
BMPR1A	AD	rs11528010	3	4.05E-01	3.64E-01	4.04E-02	B
BMPR1A	AD	rs1201398448	13	1.40E-05	1.00E-05	4.09E-02	VUS
POLE	AD	rs367970442	48	4.88E-05	4.28E-06	4.52E-02	VUS
POLE	AD	rs1060500871	47	1.40E-05	7.14E-05	3.05E-02	VUS
POLE	AD	rs200474862	43	1.40E-05	8.68E-06	2.69E-02	None
POLE	AD	rs759249822	39	None	5.47E-07	2.89E-02	None
POLE	AD	rs1301816028	38	6.98E-06	5.83E-06	4.63E-02	VUS
POLE	AD	rs148076304	37	2.09E-05	3.99E-05	9.89E-03*	VUS
POLE	AD	rs375590443	37	7.68E-05	1.16E-04	3.42E-02	VUS
POLE	AD	rs147088333	32	9.07E-05	1.35E-04	5.24E-03*	VUS
POLE	AD	rs1000912264	31	2.09E-05	2.00E-05	4.43E-02	VUS
POLE	AD	rs878854857	25	1.40E-05	1.50E-05	3.01E-02	VUS
POLE	AD	rs749012938	23	1.40E-05	4.35E-05	4.44E-02	VUS
POLE	AD	rs539312991	23	5.58E-05	3.03E-05	3.21E-02	VUS
POLE	AD	rs764044031	19	4.19E-05	1.31E-05	1.01E-02	VUS
POLE	AD	rs879254235	5	1.40E-04	5.73E-05	3.31E-02	VUS
POLE	AD	rs5744739	4	1.25E-03	3.25E-05	3.79E-03*	VUS
POLE	AD	rs879254126	1	6.99E-06	8.05E-06	2.75E-02	VUS

MLH3	AD	rs201721635	8	1.40E-04	2.40E-04	3.55E-02	VUS
MLH3	AD	rs17102999	2	9.76E-03	1.34E-02	1.84E-02	B
MLH3	AD	rs186838169	2	2.79E-05	8.00E-05	1.29E-02	None
MLH3	AD	rs61752723	2	2.93E-04	1.40E-04	3.37E-02	None
MLH3	AD	rs175080	2	4.25E-01	4.08E-01	3.97E-03*	B
MLH3	AD	rs28756982	2	1.12E-02	1.15E-02	3.18E-02	B
MLH3	AD	rs144707485	2	5.16E-04	1.36E-03	3.19E-03*	VUS
MLH3	AD	rs748434126	2	6.28E-05	2.00E-05	3.32E-02	None
MLH3	AD	rs370545907	2	4.19E-05	1.00E-05	3.80E-02	None
MLH3	AD	rs760072474	2	6.98E-06	4.00E-05	1.81E-02	VUS
BLM	AD	rs144706057	2	9.98E-04	1.27E-03	1.41E-02	VUS
BLM	AD	rs371223446	3	4.88E-05	3.00E-05	1.52E-02	VUS
BLM	AD	rs772175681	3	None	3.00E-05	1.44E-02	None
BLM	AD	rs1400231534	10	6.98E-06	4.00E-05	1.88E-02	P
BLM	AD	rs764587569	14	1.40E-05	1.00E-05	2.83E-02	VUS
BLM	AD	rs1195271909	15	2.09E-05	1.00E-05	1.52E-02	None
BLM	AD	rs370073229	16	3.49E-05	3.00E-05	3.29E-02	VUS
BLM	AD	rs202196488	16	1.26E-04	3.40E-04	1.76E-02	VUS
BLM	AD	rs730880251	21	2.09E-05	1.00E-05	4.29E-02	VUS
NTHL1	AR	rs780658029	4	1.40E-05	0	3.72E-02	None
NTHL1	AR	rs150437839	3	1.40E-05	9.00E-05	3.55E-02	None
NTHL1	AR	rs746940807	3	None	6.00E-05	2.02E-02	None
NTHL1	AR	rs369262985	2	3.49E-05	0	4.25E-02	None
NTHL1	AR	rs1422162562	2	None	0	2.71E-02	None
NTHL1	AR	rs368897948	2	1.47E-04	4.00E-05	2.14E-02	None
NTHL1	AR	rs747272786	1	6.98E-05	0	3.73E-02	None
RNF43	AD	rs1414999013	-	2.09E-05	1.00E-05	1.24E-02	None
RNF43	AD	rs9652855	9	1.13E-01	1.09E-01	2.07E-02	None
RNF43	AD	rs758947339	9	None	1.00E-05	2.87E-02	None
RNF43	AD	rs1278447549	7	6.98E-06	0	3.37E-02	None
RNF43	AD	rs2680701	7	1.37E-01	1.54E-01	4.55E-03*	None

RNF43	AD	rs200626293	6	1.47E-04	1.90E-04	2.68E-02	None
RNF43	AD	rs2257205	3	1.45E-01	1.53E-01	4.52E-03*	None
RNF43	AD	rs199909371	3	4.82E-04	8.30E-04	2.13E-02	None
RNF43	AD	rs368379005	2	1.40E-05	5.00E-05	5.16E-03*	None
SMAD4	AD	rs765597059	6	1.40E-05	3.49E-05	3.60E-02	None
STK11	AD	rs770503805	1	1.40E-05	4.00E-05	3.64E-02	VUS
STK11	AD	rs766776431	1	6.98E-06	1.00E-05	4.45E-02	None
STK11	AD	rs730881964	-	4.88E-05	6.00E-05	1.75E-02	None
STK11	AD	rs2075607	-	1.55E-01	1.99E-01	1.57E-02	None
STK11	AD	rs1034888250	8	None	0	5.13E-03*	None
STK11	AD	rs587780008	9	1.40E-05	1.00E-05	3.50E-02	VUS
STK11	AD	rs200078204	9	4.95E-04	4.50E-04	2.78E-02	VUS
STK11	AD	rs587781537	9	None	2.70E-04	2.48E-02	VUS
POLD1	AD	rs200687128	2	2.09E-05	2.10E-06	3.50E-02	None
POLD1	AD	rs946941126	4	4.19E-05	4.12E-05	4.55E-02	None
POLD1	AD	rs571623032	6	2.79E-05	2.18E-05	1.64E-02	VUS
POLD1	AD	rs1143666	6	1.21E-01	5.25E-03	1.59E-02	None
POLD1	AD	rs762330164	8	6.98E-06	3.32E-05	2.52E-02	VUS
POLD1	AD	rs773075581	10	2.79E-05	1.07E-05	2.87E-02	None
POLD1	AD	rs143076166	12	5.59E-05	1.73E-04	2.85E-03*	VUS
POLD1	AD	rs1019825673	-	1.35E-04	4.27E-05	3.10E-02	None
POLD1	AD	rs1274607	17	1.05E-01	4.16E-03	8.66E-03*	None
POLD1	AD	rs750956986	18	None	2.41E-05	3.15E-02	None
POLD1	AD	rs764432550	19	3.49E-05	3.43E-05	2.42E-02	VUS
POLD1	AD	rs149366027	20	1.37E-03	1.74E-04	1.04E-02	None
POLD1	AD	rs770495723	23	1.40E-05	1.11E-05	3.22E-02	None

Supplementary Table 3. *MUTYH* **r**ecessive analysis stratified by family history.

Recessive Analysis for those with a positive first-degree family history for CRC

MUTYH		rs34612342 (c.536A>G, Tyr179Cys)					
		TT		TC		CC	
		**Cases/ Controls**	**OR (95% CI)**	**Case/ Controls**	**OR (95% CI)**	**Cases/ Controls**	**OR (95% CI)**
rs36053993 (c.1187G>A, Gly396Asp)	CC	7543/6420	1.00 (reference)	35/26	1.31	4/1	2.03
					0.34		0.53
	CT	107/80	1.26	7/0		0 / 0	---
			0.14				
	TT	11/0		0 / 0	---	0 / 0	---

Recessive Analysis for those without a first-degree family history for CRC

MUTYH		rs34612342 (c.536A>G, Tyr179Cys)					
		TT		TC		CC	
		**Cases/ Controls**	**OR (95% CI)**	**Case/ Controls**	**OR (95% CI)**	**Cases/ Controls**	**OR (95% CI)**
rs36053993 (c.1187G>A, Gly396Asp)	CC	33266/39645	1.00 (reference)	101/117	1.086	1/0	
					0.587		
	CT	363/435	1.02	11/0		0 / 0	---
			0.789				
	TT	27/0	--	0 / 0	---	0 / 0	---

Supplementary Table 4. Comparing TOPMed and HRC (Haplotype Reference Consortium) Imputation Panel within our study population of colorectal cancer cases and controls.

MAF	TOPMed imputed		HRC imputed	
	N	Imp R2	N	Imp R2
<=0.01%	88.5M	0.42	10.3M	0.42
0.01 - 0.1%	26.4M	0.75	14.8M	0.7
0.1 - 1%	6.7M	0.87	5.7M	0.83
1 - 5%	2.6M	0.93	2.4M	0.92
>5%	6M	0.98	5.5M	0.97

Footnote Supplementary Table 3: HRC, Haplotype Reference Consortium, 32,488 samples with low coverage sequencing. TOPMed, Trans-Omics for Precision Medicine, 62,784 samples with deep coverage sequencing.

Supplementary Table 5. Testing for biallelic genetic variants to all genes involved in KEGG pathways BER, MMR and WNT (221 genes).

	**Gene**	**Variants**	**gnomad AF**	**REVEL**	**Variants**	**gnomad_AF**	**REVEL**	**OR**	**P-value**	**Estimate**	**SE**
Homozygous	EXO1	rs4149909 (p.N279S)	0.0516	0.184				1.862	0.001	0.622	0.187
	PPP3CC	p.K460R	0.009398	0.203				5.142	0.008	1.638	0.621
	BTRC	rs4151060 (p.A543S)	0.006861	0.143				1.413	0.006	0.346	0.125
Compund heterozygous	MUTYH	rs3219489 (p.Q338H)	0.4881	0.043	rs192816572 (p.A473T)	0.0001055	0.58	13.663	0.007	2.615	0.976
	RNF43	rs3744093 (p.I47V)	0.05076	0.062	rs34523089 (p.R343H)	0.05076	0.063	1.13	0.005	0.122	0.044
	SFRP4	rs185762400 (c.269+3614C>G	0.00438	-	rs186132418 (c.-53+28980A>T	0.00252	-	6.601	0.036	1.887	0.9
	HMGB1	rs150085905 (c.-14-33206C>T	0.02	-	rs148916860 (c.-15+43878G>A	0.047	-	3.472	0.037	1.245	0.597
	FRAT2	rs528434717 (p.A56T)	0.007189	0.05	rs561034462 (p.A56T)	0.007189	0.03	5.312	0.051	1.67	0.856
	HMGB1	rs7988478 (c.-14-33152T>C)	0.0345	-	rs148916860 (c.-15+43878G>A	0.00477	-	4.264	0.057	1.45	0.762
	PARP4	rs147633033 (p.L340P)	0.004848	0.206	rs1050114 (p.A1331T)	0.1645	0.017	5.825	0.065	1.762	0.954
	LGR4	rs75496259 (p.T327A)	0.004848	0.116	rs34717439 (p.T709M)	0.004848	0.157	6.302	0.069	1.841	1.012
	PARP4	rs35200240 (p.I81V)	0.004848	0.151	rs145290616 (p.G496V)	0.004848	0.458	3.326	0.07	1.202	0.663
	LGR5	rs10879296 (c.1070+404G>C)	0.005866	-	rs73339868 (p.S435L)	0.007189	0.232	5.237	0.074	1.656	0.927
	PARP4	rs116847311 (p.V169G)	0.0477	0.112	rs147633033 (p.L340P)	0.004848	0.206	4.934	0.081	1.596	0.915
	NKD2	rs144426465 (p.A72T)	0.1619	0.033	rs141899564 (p.M144K)	0.1619	0.266	6.551	0.086	1.88	1.093
	PMS2	rs1805324 (p.M622I)	0.5799	0.09	rs372341850 (p.R646M)	0.5799	0.373	9.653	0.087	2.267	1.325

Supplementary Table 6. Genes for pathway analysis derived from the KEGG pathways BER, MMR and WNT (221 genes).

**Baser excision repair**		**MMR**		**WNT signaling**	
OGG1	8-oxoguanine DNA glycosylase	SSBP1	single stranded DNA binding protein 1	PORCN	porcupine O-acyltransferase
NTHL1	nth like DNA glycosylase 1	PMS2	PMS1 homolog 2, mismatch repair system componer	WNT1	Wnt family member 1
NEIL1	nei like DNA glycosylase 1	MLH1	mutL homolog 1	WNT2	Wnt family member 2
NEIL2	nei like DNA glycosylase 2	MSH6	mutS homolog 6	WNT2B	Wnt family member 2B
NEIL3	nei like DNA glycosylase 3	MSH2	mutS homolog 2	WNT3	Wnt family member 3
UNG	uracil DNA glycosylase	MSH3	mutS homolog 3	WNT3A	Wnt family member 3A
SMUG1	single-strand-selective monofunctional uracil-DNA glycosylase	MLH3	mutL homolog 3	WNT4	Wnt family member 4
MUTYH	mutY DNA glycosylase	RFC1	replication factor C subunit 1	WNT5A	Wnt family member 5A
MPG	N-methylpurine DNA glycosylase	RFC4	replication factor C subunit 4	WNT5B	Wnt family member 5B
MBD4	methyl-CpG binding domain 4, DNA glycosylase	RFC2	replication factor C subunit 2	WNT6	Wnt family member 6
TDG	thymine DNA glycosylase	RFC5	replication factor C subunit 5	WNT7A	Wnt family member 7A
APEX1	apurinic/apyrimidinic endodeoxyribonuclease 1	RFC3	replication factor C subunit 3	WNT7B	Wnt family member 7B
PNKP	polynucleotide kinase 3'-phosphatase	PCNA	proliferating cell nuclear antigen	WNT8A	Wnt family member 8A
TDP1	tyrosyl-DNA phosphodiesterase 1	EXO1	exonuclease 1	WNT8B	Wnt family member 8B
POLB	DNA polymerase beta	RPA1	replication protein A1	WNT9A	Wnt family member 9A
POLL	DNA polymerase lambda	RPA2	replication protein A2	WNT9B	Wnt family member 9B
HMGB1	high mobility group box 1	RPA3	replication protein A3	WNT10B	Wnt family member 10B
PARP1	poly(ADP-ribose) polymerase 1	RPA4	replication protein A4	WNT10A	Wnt family member 10A
PARP2	poly(ADP-ribose) polymerase 2	POLD1	DNA polymerase delta 1, catalytic subunit	WNT11	Wnt family member 11
PARP3	poly(ADP-ribose) polymerase family member 3	POLD2	DNA polymerase delta 2, accessory subunit	WNT16	Wnt family member 16
PARP4	poly(ADP-ribose) polymerase family member 4	POLD3	DNA polymerase delta 3, accessory subunit	CER1	cerberus 1, DAN family BMP antagonist
PARG	poly(ADP-ribose) glycohydrolase	POLD4	DNA polymerase delta 4, accessory subunit	NOTUM	notum, palmitoleoyl-protein carboxylesterase
ADPRS	ADP-ribosylserine hydrolase	LIG1	DNA ligase 1	WIF1	WNT inhibitory factor 1
APTX	aprataxin			SOST	sclerostin
XRCC1	X-ray repair cross complementing 1			DKK1	dickkopf WNT signaling pathway inhibitor 1
POLG	DNA polymerase gamma, catalytic subunit			DKK2	dickkopf WNT signaling pathway inhibitor 2
POLG2	DNA polymerase gamma 2, accessory subunit			DKK4	dickkopf WNT signaling pathway inhibitor 4
LIG3	DNA ligase 3			SERPINF1	serpin family F member 1
POLD1	DNA polymerase delta 1, catalytic subunit			SFRP1	secreted frizzled related protein 1
POLD2	DNA polymerase delta 2, accessory subunit			SFRP2	secreted frizzled related protein 2
POLD3	DNA polymerase delta 3, accessory subunit			FRZB	frizzled related protein
POLD4	DNA polymerase delta 4, accessory subunit			SFRP4	secreted frizzled related protein 4
POLE	DNA polymerase epsilon, catalytic subunit			SFRP5	secreted frizzled related protein 5
POLE2	DNA polymerase epsilon 2, accessory subunit			RSPO1	R-spondin 1
POLE3	DNA polymerase epsilon 3, accessory subunit			RSPO2	R-spondin 2
POLE4	DNA polymerase epsilon 4, accessory subunit			RSPO3	R-spondin 3
PCNA	proliferating cell nuclear antigen			RSPO4	R-spondin 4
RFC1	replication factor C subunit 1			LGR4	leucine rich repeat containing G protein-coupled receptor 4
RFC4	replication factor C subunit 4			LGR5	leucine rich repeat containing G protein-coupled receptor 5
RFC2	replication factor C subunit 2			LGR6	leucine rich repeat containing G protein-coupled receptor 6
RFC5	replication factor C subunit 5			RNF43	ring finger protein 43
RFC3	replication factor C subunit 3			ZNRF3	zinc and ring finger 3
FEN1	flap structure-specific endonuclease 1			FZD1	frizzled class receptor 1
LIG1	DNA ligase 1			FZD7	frizzled class receptor 7
				FZD2	frizzled class receptor 2
				FZD3	frizzled class receptor 3
				FZD4	frizzled class receptor 4
				FZD5	frizzled class receptor 5
				FZD8	frizzled class receptor 8
				FZD6	frizzled class receptor 6
				FZD10	frizzled class receptor 10
				FZD9	frizzled class receptor 9
				LRP5	LDL receptor related protein 5
				LRP6	LDL receptor related protein 6
				BAMBI	BMP and activin membrane bound inhibitor
				APCDD1	APC down-regulated 1
				APCDD1L	APC down-regulated 1 like
				CSNK1E	casein kinase 1 epsilon
				TPTEP2-CSNK1E	TPTEP2-CSNK1E readthrough
				CCDC88C	coiled-coil domain containing 88C
				DVL3	dishevelled segment polarity protein 3
				DVL2	dishevelled segment polarity protein 2
				DVL1	dishevelled segment polarity protein 1
				FRAT1	FRAT regulator of WNT signaling pathway 1
				FRAT2	FRAT regulator of WNT signaling pathway 2
				CSNK2A1	casein kinase 2 alpha 1
				CSNK2A2	casein kinase 2 alpha 2
				CSNK2A3	casein kinase 2 alpha 3
				CSNK2B	casein kinase 2 beta
				NKD1	NKD inhibitor of WNT signaling pathway 1
				NKD2	NKD inhibitor of WNT signaling pathway 2
				CXXC4	CXXC finger protein 4
				SENP2	SUMO specific peptidase 2
				GSK3B	glycogen synthase kinase 3 beta
				CTNNB1	catenin beta 1
				AXIN1	axin 1
				AXIN2	axin 2
				APC	APC regulator of WNT signaling pathway
				APC2	APC regulator of WNT signaling pathway 2
				CSNK1A1L	casein kinase 1 alpha 1 like
				CSNK1A1	casein kinase 1 alpha 1
				TCF7	transcription factor 7
				TCF7L1	transcription factor 7 like 1
				TCF7L2	transcription factor 7 like 2
				LEF1	lymphoid enhancer binding factor 1
				CTNNBIP1	catenin beta interacting protein 1
				CBY1	chibby family member 1, beta catenin antagonist
				CHD8	chromodomain helicase DNA binding protein 8
				SOX17	SRY-box transcription factor 17
				CTBP1	C-terminal binding protein 1

				CTBP2	C-terminal binding protein 2
				TLE7	TLE family member 7
				TLE1	TLE family member 1, transcriptional corepressor
				TLE2	TLE family member 2, transcriptional corepressor
				TLE3	TLE family member 3, transcriptional corepressor
				TLE4	TLE family member 4, transcriptional corepressor
				TLE6	TLE family member 6, subcortical maternal complex member
				CTNND2	catenin delta 2
				CREBBP	CREB binding protein
				EP300	E1A binding protein p300
				RUVBL1	RuvB like AAA ATPase 1
				SMAD4	SMAD family member 4
				SMAD3	SMAD family member 3
				MAP3K7	mitogen-activated protein kinase kinase kinase 7
				NLK	nemo like kinase
				MYC	MYC proto-oncogene, bHLH transcription factor
				JUN	Jun proto-oncogene, AP-1 transcription factor subunit
				FOSL1	FOS like 1, AP-1 transcription factor subunit
				CCND1	cyclin D1
				CCND2	cyclin D2
				CCND3	cyclin D3
				CCN4	cellular communication network factor 4
				PPARD	peroxisome proliferator activated receptor delta
				MMP7	matrix metallopeptidase 7
				PSEN1	presenilin 1
				PRKACA	protein kinase cAMP-activated catalytic subunit alpha
				PRKACB	protein kinase cAMP-activated catalytic subunit beta
				PRKACG	protein kinase cAMP-activated catalytic subunit gamma
				TP53	tumor protein p53
				SIAH1	siah E3 ubiquitin protein ligase 1
				CACYBP	calcyclin binding protein
				SKP1	S-phase kinase associated protein 1
				TBL1X	transducin beta like 1 X-linked
				TBL1Y	transducin beta like 1 Y-linked
				TBL1XR1	TBL1X/Y related 1
				BTRC	beta-transducin repeat containing E3 ubiquitin protein ligase
				FBXW11	F-box and WD repeat domain containing 11
				CUL1	cullin 1
				RBX1	ring-box 1
				GPC4	glypican 4
				ROR1	receptor tyrosine kinase like orphan receptor 1
				ROR2	receptor tyrosine kinase like orphan receptor 2
				RYK	receptor like tyrosine kinase
				VANGL2	VANGL planar cell polarity protein 2
				VANGL1	VANGL planar cell polarity protein 1
				PRICKLE1	prickle planar cell polarity protein 1
				PRICKLE2	prickle planar cell polarity protein 2
				PRICKLE4	prickle planar cell polarity protein 4
				PRICKLE3	prickle planar cell polarity protein 3
				INVS	inversin
				DAAM1	dishevelled associated activator of morphogenesis 1
				DAAM2	dishevelled associated activator of morphogenesis 2
				RHOA	ras homolog family member A
				ROCK2	Rho associated coiled-coil containing protein kinase 2
				RAC1	Rac family small GTPase 1
				RAC2	Rac family small GTPase 2
				RAC3	Rac family small GTPase 3
				MAPK8	mitogen-activated protein kinase 8
				MAPK10	mitogen-activated protein kinase 10
				MAPK9	mitogen-activated protein kinase 9
				PLCB1	phospholipase C beta 1
				PLCB2	phospholipase C beta 2
				PLCB3	phospholipase C beta 3
				PLCB4	phospholipase C beta 4
				CAMK2A	calcium/calmodulin dependent protein kinase II alpha
				CAMK2D	calcium/calmodulin dependent protein kinase II delta
				CAMK2B	calcium/calmodulin dependent protein kinase II beta
				CAMK2G	calcium/calmodulin dependent protein kinase II gamma
				PPP3CA	protein phosphatase 3 catalytic subunit alpha
				PPP3CB	protein phosphatase 3 catalytic subunit beta
				PPP3CC	protein phosphatase 3 catalytic subunit gamma
				PPP3R1	protein phosphatase 3 regulatory subunit B, alpha
				PPP3R2	protein phosphatase 3 regulatory subunit B, beta
				PRKCA	protein kinase C alpha
				PRKCB	protein kinase C beta
				PRKCG	protein kinase C gamma
				NFATC1	nuclear factor of activated T cells 1
				NFATC2	nuclear factor of activated T cells 2
				NFATC3	nuclear factor of activated T cells 3
				NFATC4	nuclear factor of activated T cells 4
