# Supplementary material for: Elucidating the Risk of Colorectal Cancer for Variants in Hereditary Colorectal Cancer Genes
Source: Gastroenterology. Author manuscript; Available in PMC 2024 Feb 14. (PMC10866455; doi:10.1053/j.gastro.2023.06.032)
Supplement: 2 [file NIHMS1962932-supplement-2.pdf]

Supplementary Material

Study Population

We combined studies from 3 consortia: the Genetics and Epidemiology of Colorectal Cancer Consortium (GECCO), the Colorectal Cancer Transdisciplinary Study (CORECT), and the Colon Cancer Family Registry (CCFR). Most studies have been described in detail previously,[1–4] and additional studies not previously included are described in detail here. After quality control, we included 58,920 CRC or advanced adenoma case individuals and 71,171 control individuals of European ancestry. We confirmed genetic ancestry by principal component analysis[5,6] on a shared, linkage disequilibrium–pruned set of autosomal single-nucleotide variants and comparison with 1092 individuals from the 1000 Genomes Project.[7] Supplementary Table 1 provides details on the sample numbers and demographic characteristics of the study participants. All participants provided written informed consent, and each study was approved by the relevant research ethics committee or institutional review board.

Genome-Wide Association Study Genotype Data and Imputation

Details of genotyping and quality control for studies included are described elsewhere,[1–4] and samples not previously included underwent quality control analysis using standardized methods as detailed in Laurie et al.[5] The genotyping data were imputed to the National Heart, Lung, and Blood Institute TOPMed Consortium version r1 panel using the University of Michigan Imputation Server.[8] The TOPMed panel comprises whole-genome sequencing data from 97,256 samples and 308,107,085 genetic variants. To improve imputation accuracy and phasing, imputation was performed after pooling studies or genotyping projects that used the same or very similar genotyping platforms (Supplementary Table 1).

Statistical Analysis

We performed a genome-wide association analysis of individual variants with CRC risk using the score test under the log-additive logistic regression model, adjusting for sex, age, and 20 principal components to account for population substructure. Stratifying by genotyping platform, we calculated the score statistic and corresponding variance for each variant. We then combined the platform-specific results by summing score statistics U and variance V and obtained the study-wide P value based on the score test $U/V^{1/2}$.[9]

In addition to genome-wide analysis, we examined the association of variants (MAF $<$ 1%) in 22 moderate- to high-penetrance genes that were previously reported to be established or with suggestive evidence for association with CRC risk.[10] This set of genes includes 16 consensus hereditary CRC and polyposis risk genes (mismatch repair genes) (*MLH1*, *MSH2*, *MSH6*, *PMS2*, *EPCAM*, *APC*, *BLM*, *BMPR1A*, *GREM1*, *MUTYH*, *NTHL1*, *POLD1*, *POLE*, *PTEN*, *SMAD4*, and *STK11*) and 6 genes with accumulating evidence for association with CRC and/or polyposis predisposition (*AXIN2*, *MSH3*, *MLH3*, *RNF43*, *MBD4*, and *RPS20*).[10–14]

In addition to the log-additive association test, we assessed the association analysis under a recessive model for *MUTYH* because this gene is known to have a recessive mode of inheritance.[11] In addition, we examined the association of biallelic carriers (compound heterozygotes and homozygous carriers) for pairwise variants within *MUTYH*, *NTHL1*, *MSH3*, and *MBD4* genes. Because the frequency of biallelic carriers is rare, we pooled individual-level data from all samples for the analysis and ran the logistic regression model adjusting for age, sex, and genotyping platform rather than performing a meta-analysis.

Finally, we performed aggregate tests at the gene level using our set-based MiST.[15] MiST tests the association of the total effect of the variants in a set by combining the burden component test that accounts for functional annotations of variants and the variance component test for the associations of individual variants that have not been explained by the burden components using the Fisher combination test. We incorporated in silico functional effect prediction scores from CADD[16,17] and REVEL[18] and population frequency from gnomAD (version 3.1)[19] as weights to calculate the weighted burden scores. MiST performs among the best in terms of statistical power across a range of architectures[20] and has recently been extended to using summary statistics only.[21] We used the score statistics as the summary statistics and individual-level genotyping data (n $=$ 8725) from GECCO to estimate linkage disequilibrium. Variants with a minor allele count of $<$10 or imputation R^2 of $<$0.3 were excluded.

For genome-wide marginal and set-based analyses, we used Bonferroni correction to account for multiple comparisons and considered a 2-sided P value of $<5 \times 10^{-8}$ and $<$0.05/22 genes $=$ 0.0024 as statistically significant, respectively. For all other analyses, we considered a 2-sided P value of $<$.05 as showing evidence for association.

Variant Annotation

Variants were annotated, and their biological consequences were determined using Ensembl Variant Effect Predictor (version 94).[22] Functional impact on gene function was predicted for missense variants using in silico tools PolyPhen-2,[23] SIFT,[24,25] CADD,[16,17] and REVEL.[18] Variants with CADD of $>$20 or REVEL of $>$0.5 were considered to be "predicted pathogenic." The variants were mapped to the ClinVar database[26] to incorporate ClinVar classifications. Population level variant allele frequencies were obtained from the population-based reference dataset, gnomAD (version 3.0), where the variants are derived from a harmonized dataset of 71,702 whole-genome sequences.[19]

Additional Genes for Recessive Analysis

Recessive genes *MUTYH*, *NTHL1*, *MSH3*, and *MBD4* are associated with CRC and colonic polyposis. We did not observe that heterozygous carriers of a single pathogenic

variant in these genes are associated with an increased risk of CRC; therefore, increased screening for CRC may not be warranted in these individuals. Further, given the varying frequency of these *MUTYH* variants in specific Jewish populations (North African/Moroccan Jews), reanalysis excluding samples from the study conducted in Israel showed similar associations as shown in Table 1 (data not shown).

Similarly, monoallelic carriers of a pathogenic/likely pathogenic variant in the recessively inherited CRC genes *NTHL1*, *MSH3*, and *MBD4* showed no evidence of an increased risk of CRC. We could not test for CRC risks for biallelic (homozygous or compound heterozygous) carriers for the *NTHL1* variants c.268C>T p.Gln90Ter (rs150766139; P = .54; MAF, 0.17%) and c.859C>T p.Gln287Ter (rs146347092; P = .6; MAF, 0.02%) because only a single biallelic carrier was observed in the dataset. No biallelic *MSH3* or *MBD4* carriers were observed.

Recurrent High-Risk Variants

Investigation of recurrent, high-risk variants in other cancer genes found no evidence for an association with CRC risk for the prostate cancer risk allele in *HOXB13* (c.251G>A, p.Gly84Glu, rs138213197; MAF, 0.22%; P = .47) or the melanoma risk allele in *MITF* (c.1273G>A, p.Glu318Lys, rs149617956; MAF, 0.14%; P = .53). We were not able to impute the *CHEK2* c.1100delC p.Thr367fs (rs555607708) and *ATM* c.7271T>G p.Val2424Gly (rs28904921) variants. The *CHEK2* c.470T>C p.Ile157Thr (rs17879961)[27] variant was imputed; however, no significant association was observed.

Biallelic Assessment of Candidate Colorectal Cancer Pathway Genes

An additional 221 genes (Supplementary Table 6) were sourced from the CRC-associated base excision repair, mismatch repair, and Wnt-signaling (Wingless/integrated) KEGG (Kyoto Encyclopedia of Genes and Genomes) pathways to expand our investigation into recessive acting genes (either homozygous or compound heterozygote). For this, we selected all nonsynonymous variants with an MAF of <5% and >10 minor allele count within a gene and tested either for homozygous or compound heterozygous carriers of all variant combinations. Despite the expansive evaluation, we did not identify any additional recessive acting gene after accounting for multiple comparisons (Supplementary Table 5).

Supplementary References

1. Huyghe JR, Bien SA, Harrison TA, et al. Nat Genet 2019;51:76–87.
2. Peters U, Jiao S, Schumacher FR, et al. Gastroenterology 2013;144:799–807.e24.
3. Schmit SL, Edlund CK, Schumacher FR, et al. J Natl Cancer Inst 2019;111:146–157.
4. Schumacher FR, Schmit SL, Jiao S, et al. Nat Commun 2015;6:7138.
5. Laurie CC, Doheny KF, Mirel DB, et al. Genet Epidemiol 2010;34:591–602.
6. Price AL, Weale ME, Patterson N, et al. Am J Hum Genet 2008;83:132–135; author reply 135–139.
7. Consortium GP, Abecasis GR, Altshuler D, et al. Nature 2010;467:1061–1073.
8. Taliun D, Harris DN, Kessler MD, et al. Nature 2021;590:290–299.
9. Liu DJ, Peloso GM, Zhan X, et al. Nat Genet 2014;46:200–204.
10. Seifert BA, McGlaughon JL, Jackson SA, et al. Genet Med 2019;21:1507–1516.
11. Belhadj S, Terradas M, Munoz-Torres PM, et al. Hum Mutat 2020;41:1563–1576.
12. Grolleman JE, de Voer RM, Elsayed FA, et al. Cancer Cell 2019;35:256–266.e5.
13. Palles C, West HD, Chew E, et al. Am J Hum Genet 2022;109:953–960.
14. Weren RD, Ligtenberg MJ, Kets CM, et al. Nat Genet 2015;47:668–671.
15. Sun J, Zheng Y, Hsu L. Genet Epidemiol 2013;37:334–344.
16. Kircher M, Witten DM, Jain P, et al. Nat Genet 2014;46:310–315.
17. Rentzsch P, Witten D, Cooper GM, et al. Nucleic Acids Res 2019;47:D886–D894.
18. Ioannidis NM, Rothstein JH, Pejaver V, et al. Am J Hum Genet 2016;99:877–885.
19. Karczewski KJ, Francioli LC, Tiao G, et al. Nature 2020;581:434–443.
20. Moutsianas L, Agarwala V, Fuchsberger C, et al. PLoS Genet 2015;11:e1005165.
21. Dong X, Su YR, Barfield R, et al. PLoS Genet 2020;16:e1008947.
22. McLaren W, Gil L, Hunt SE, et al. Genome Biol 2016;17:122.
23. Adzhubei IA, Schmidt S, Peshkin L, et al. Nat Methods 2010;7:248–249.
24. Kumar P, Henikoff S, Ng PC. Nat Protoc 2009;4:1073–1081.
25. Ng PC, Henikoff S. Genome Res 2001;11:863–874.
26. Landrum MJ, Lee JM, Benson M, et al. Nucleic Acids Res 2018;46:D1062–D1067.
27. Rohlin A, Rambech E, Kvist A, et al. Fam Cancer 2017;16:195–203.
